# Ethanol-Assisted Alkanethiol Self-Assembled Monolayer Disruption by Mobile Siloxane Oligomers for Precise Galvanic Replacement Positioning

**DOI:** 10.1021/acsami.5c23948

**Published:** 2026-02-17

**Authors:** Yu-Ling Tu, Chia-Li Liao, Elmer Ismael Guerra, Heng-Yu Yang, Yu-Chieh Wen, Lee-Chiang Lo, Wei-Ssu Liao

**Affiliations:** † Department of Chemistry, 33561 National Taiwan University, Taipei 10617, Taiwan; ‡ Center for Emerging Material and Advanced Devices, 33561 National Taiwan University, Taipei 10617, Taiwan; § Institute of Physics, Academia Sinica, Taipei 11529, Taiwan

**Keywords:** self-assembled monolayer, Au-thiolate, siloxane oligomer, galvanic replacement, metal-enhanced fluorescence

## Abstract

Siloxane oligomers with low molecular weights often exist in elastomeric polymers, e.g., polydimethylsiloxane, and can be troublesome chemical species when utilizing polymers due to their hard-to-control behavior and unpredictable mobility. For instance, the presence of these species can contaminate surfaces and affect molecular integrity when these polymers are applied in developing functional substrates. In this study, on the contrary, we provide an unconventional approach whereby siloxane oligomers originating from a polymerized matrix are transported to alkanethiol self-assembled monolayer (SAM)-functionalized Au through the mediation of ethanol pre-entrapped in the elastomer. Relying on the interface mobile environment provided by ethanol, siloxane oligomers controllably diffuse, transfer, and disrupt a preformed alkanethiol monolayer on Au during conformal contact sealing, which in turn promotes the detachment of Au-thiolates from the surface. Spectroscopic analyses, including sum frequency generation vibrational spectroscopy and X-ray photoelectron spectroscopy, confirm the disruption of SAMs and the detachment of Au-thiolates. Several key parameters, including conformal contact sealing duration, molecule backbone chain length, and terminal group functionality, are critical in this SAM disruption phenomenon. The produced disordered SAM environment enables the penetration of ions when placed in solutions and supports underlying metal oxidation for precise feature transfer. Furthermore, selective galvanic replacements between different metals can be triggered at SAM-disrupted regions to produce bimetallic substrates. These bimetallic interfaces selectively enhance fluorophore-dependent fluorescence emission by interparticle electric field promotion. By combining multiple spatial SAM disruption operations on the same substrate, the produced fluorescent assay, built with manifold internal standards, offers a reliable platform, supporting ratiometric treatments for further bioimaging analysis and detection.

## Graphical abstract

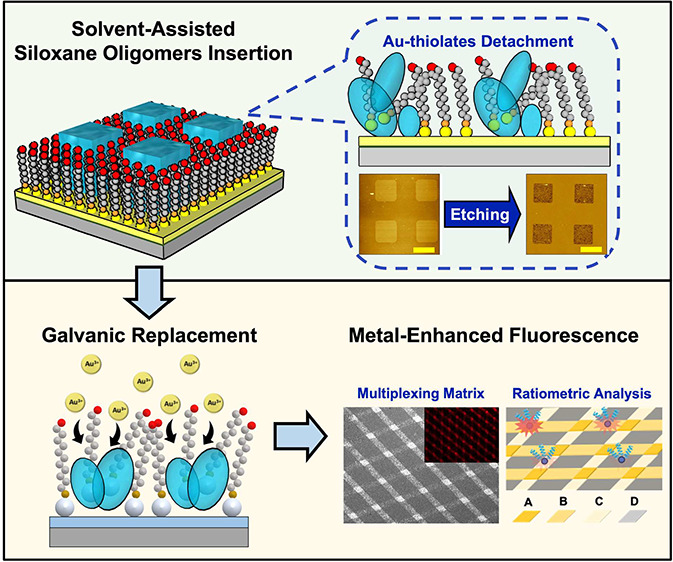

## Introduction

Delicate control over molecular orientation, positioning, and arrangement on a surface enables the generation of functional substrates for a variety of physical, biological, and engineering applications.^1–5^ The demands of modern sensor and semiconductor chip designs have particularly driven inventions of different approaches in creating adjustable molecular systems and high-resolution patterns on solid supports. To achieve an energetic, stable, and robust interface for these objects, selecting compatible molecule/surface pairs, introducing appropriate operation strategies, and employing a suitable chemical environment are critical issues.

Self-assembled monolayer (SAM) systems, which rely on spontaneous molecule arrangement in an energy-favorable manner, play a crucial role in contemporary nanotechnology.^6,7^ Due to their ease of preparation and compatibility toward numerous substrates,^8–10^ they are widely adapted in the fields of corrosion protection,^11^ biosensor design,^12^ electronic device assembly,^13,14^ and micro/nano fabrication.^10,15^ Two of the most commonly employed SAM/surface combinations include thiol and silane chemistry on noble metal and silicon substrates, respectively. With thiol chemistry, the SAM forms on a transition metal surface through strong chemical bonds between the alkanethiol headgroups and the substrate. This monolayer is stabilized by van der Waals interactions between hydrocarbon chains of neighboring molecules, resulting in a well-ordered crystalline alkanethiolate film. By adjusting the terminal group of alkanethiol molecules, the physicochemical properties of the treated surface can also be controlled to meet different demands.^10,16^ Comparably, silane chemistry involves the reaction between silanes and hydroxylated surfaces.^17,18^ These well-ordered and dense monolayers provide high chemical stability due to the formation of multiple bonds between their anchoring groups and the surface. Additionally, their terminal groups can be further modified to customize the exposed surface properties.^7^

In addition to suitable molecule/surface pair selection, techniques that can provide precise chemical functionality positioning and array generation are useful for practical molecular matrix creation. The key to achieving molecular manipulation and boundary control depends on constraining interface intermolecular forces to regulate high-resolution feature integrity.^19^ This demand has led to the invention of molecular patterning strategies that can provide feature dimensions down to the nanometer scale or even the single molecule level.^20,21^ The existing techniques can be broadly categorized into two main types: spontaneous self-assembly and guided pattern creation. In self-assembly approaches, molecules spontaneously organize into ordered structures driven by molecule–substrate interactions or intermolecular forces. Representative examples of molecular self-assembly include covalent bonding-driven alkanethiol SAMs on metals,^10,16^ intermolecular hydrogen bonding-guided assembly,^22^ and electron donor–acceptor interactions.^23^ These self-assembly strategies highlight the importance of molecular interactions in directing the formation of well-organized films, offering powerful routes for designing advanced functional materials. In comparison, guided pattern creation generates molecular patterns dependent on external processing to selectively remove or insert molecules for controlling molecular arrangements.^24,25^ Representative techniques include photolithography,^21^ electron beam lithography,^26^ scanning probe lithography,^27,28^ and soft lithography.^19^ Although these approaches all present privileges with drawbacks, appropriate process modifications and technique combinations can be applied to solve their limitations based on needs.

Taking the aforementioned soft lithographic printing techniques as examples, performing molecular operations in a compatible chemical environment is the critical consideration when a well-defined molecular matrix is desired. Soft lithographic techniques transfer molecules using elastomeric stamps, offering a simple, low-cost, and high-throughput approach to generate well-ordered molecular patterns with controllable dimensions.^19^ An efficient transfer of molecular inks from a rubber stamp to the target surface requires an environment that can provide sufficient mobility for ink molecules without severe lateral diffusion.^29^ Conventionally, conformal contact between a stamp and a substrate in the printing process is conducted under ambient conditions. The printed pattern dimension and integrity rely on the polarities of both the ink and the substrate, as well as characteristics of the molecular transport medium. Although these molecular inking processes under ambient conditions realize numerous successful patterning approaches, the involvement of organic solvents in the procedure can provide further operational parameters that may enable better control over molecular movement.^30,31^ Nevertheless, challenges such as stamp deformation, swelling, and the presence of uncross-linked low-molecular-weight (LMW) moieties may compromise precise patterning control.^31,32^ These factors reduce feature resolution, pattern reproducibility, and may introduce impurities onto the substrate during the printing process.^31^ For instance, commonly used PDMS stamps may encounter deformation, buckling, and lateral collapse when a solvent is introduced in the printing procedure.^31,33^ Besides, the selection of distinct solvents may result in different levels of PDMS swelling, thus affecting the final printing resolution.^31,34^ Furthermore, uncross-linked LMW siloxanes contained within the PDMS stamp could contaminate and degrade the quality of printed patterns on the surface.^31,35^ These factors thus ultimately limit the printed structure precision and feature reproducibility when a solvent is involved.

In this work, we present an unconventional strategy that leverages LMW siloxane oligomers inside PDMS, with ethanol as a mediator, to achieve controllable alkanethiol SAM disruption. This unique combination enables a molecular patterning technique that disrupts the well-ordered alkanethiol SAM and facilitates the detachment of interface Au-thiolates. Under this operation, initial PDMS-substrate contact results in protruding features made up of LMW siloxane oligomers. Surprisingly, these protruding features become depressed metal structures following a wet chemical etching process. Surface-sensitive spectroscopic techniques, including sum frequency generation-vibrational spectroscopy (SFG-VS) and X-ray photoelectron spectroscopy (XPS), point to the weakened bond energy between Au-thiolates and their neighboring Au atoms. This is attributed to the oxygen-rich nature of siloxane oligomers, which allows for electron attraction from surface-bound Au atoms.^36,37^ Parameters such as PDMS sealing time, thiol chain length, and molecule terminal groups are key factors affecting this molecular matrix disruption process. Utilizing the disrupted alkanethiol SAM matrix, spatially selective galvanic replacements between two metals at this unique interface environment are achievable. The introduced metal ions can successfully penetrate the disrupted SAM region and replace the original metal atoms underneath to produce distribution-controllable bimetallic surfaces. Our results demonstrate that these precisely engineered bimetallic substrates enable the fluorophore-selective metal-enhanced fluorescence (MEF) phenomenon. The integration of designed multiplexed substrates with optically compatible fluorescent dyes thus realizes multilevel precise sample detections through ratiometric imaging analysis.

## Results and Discussion

### Disruption of Alkanethiol SAMs and Detachment of Au-Thiolates by an Ethanol-Assisted Process

The selective disruption of an ordered SAM through LMW moieties relies on the synergistic combination of a robust alkanethiol/Au pair, the PDMS-substrate conformal contact for effective molecular transfer, and an ethanol-mediated environment that facilitates molecular mobility. Instead of removing uncross-linked siloxane oligomers, referred to as PDMS residues, in a conformal contact sealing process here, these moieties are intentionally transferred onto 11-Mercaptoundecanol (MCU)-covered Au in this operation (Scheme 1). With the assistance of ethanol pre-entrapped in PDMS, LMW siloxane oligomers retain sufficient mobility to diffuse out of the cross-linked PDMS structure and deposit onto the MCU-covered Au. During the conformal sealing process, the oxygen-rich siloxane oligomers weaken the bond energy between alkanethiol-attached Au and neighboring Au atoms by attracting electrons. Meanwhile, the interface environment rich with ethanol molecules provides sufficient mobility for Au-thiolates and eventually promotes their detachment from the Au substrate. Simultaneously, the required coexistence of both mobile ethanol and siloxane oligomer molecules is restricted at contact areas, which confines the interfered SAM region and preserves the patterned feature boundary and integrity. This process thus leads to the spatially controlled insertion of siloxane oligomers into the well-ordered SAM at the contact region, where the local alkanethiol molecular matrix is severely disturbed.

**Scheme 1:**
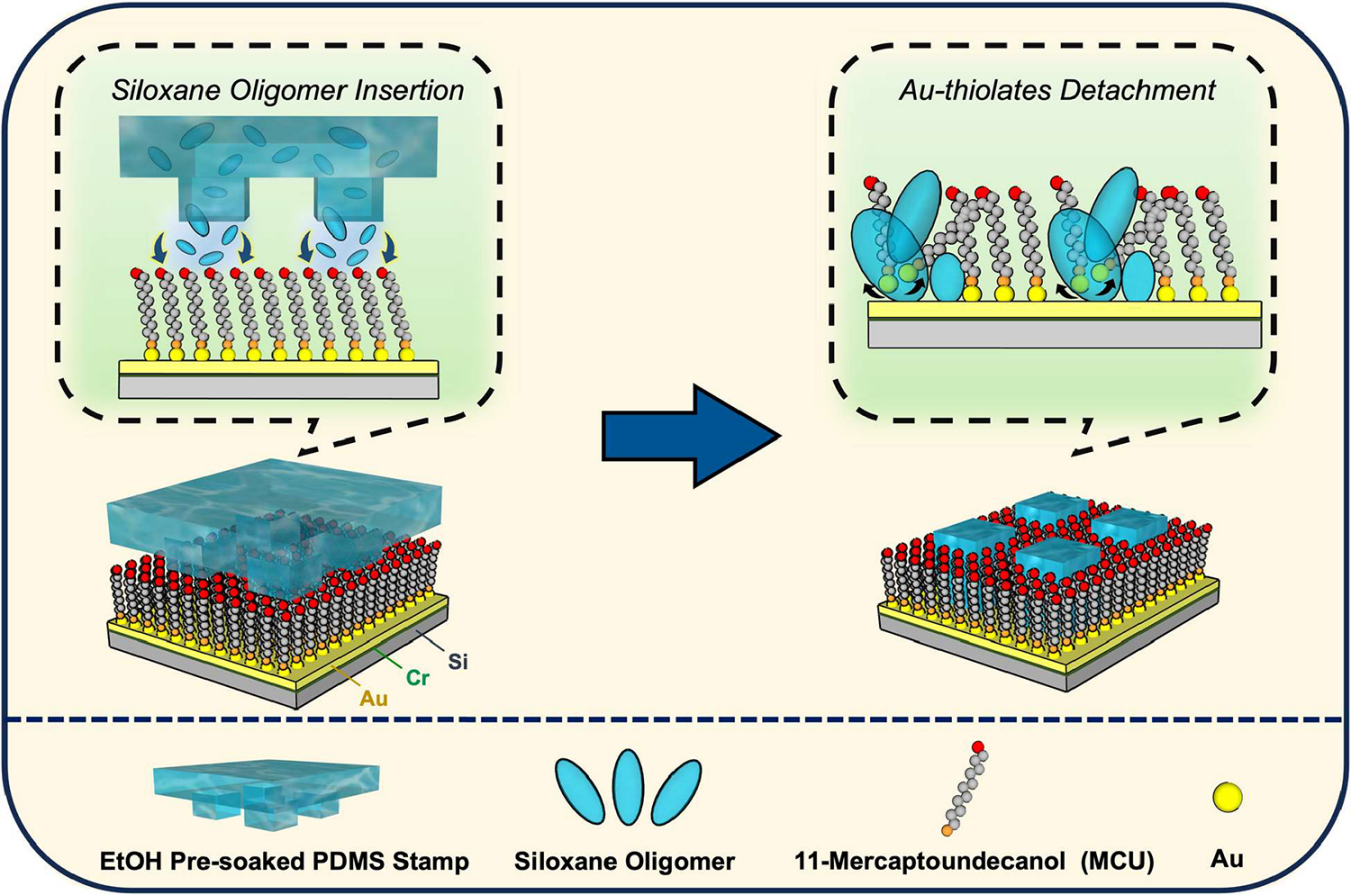
Illustration of the Solvent-Assisted Insertion of Mobile Siloxane Oligomers onto a MCU SAM-Covered Au Substrate and the Resulting Au-Thiolate Detachment during the Conformal Contact Sealing Process

To investigate the interface molecular environment changes during the ethanol-assisted SAM disruption by siloxane oligomers, XPS was used to systematically study the electronic state of Au throughout this process. As shown in Figure 1A, the Au 4f_7/2_ and Au 4f_5/2_ peaks are found at 83.78 and 87.45 eV for bare Au (Figure 1A­(i)), while MCU-covered Au (Figure 1A­(ii)) gives peaks at 83.90 and 87.57 eV, respectively. The higher binding energy of Au 4f in the MCU-covered Au suggests the formation of Au–S bonds, which is consistent with previous studies.^10^ After treatment by an ethanol-presoaked PDMS stamp conformal sealing (Figure 1A­(iii)), the Au 4f binding energy shifts lower to 83.71 eV (Au 4f_7/2_) and 87.38 eV (Au 4f_5/2_), close to that of bare Au. In contrast, the Au 4f_7/2_ and Au 4f_5/2_ peaks for the MCU-covered Au, after an identical PDMS stamping procedure without ethanol presoaking, are found at 83.89 and 87.56 eV, respectively (Figure 1A­(iv)). There is essentially no significant change found in the Au 4f binding energy when compared to the original MCU-covered Au surface. This observation not only indicates an obvious reduction in Au–S bonds resulting from the ethanol-assisted conformal contact sealing process but also highlights the essential role of ethanol in enabling this phenomenon.

**Figure 1:**
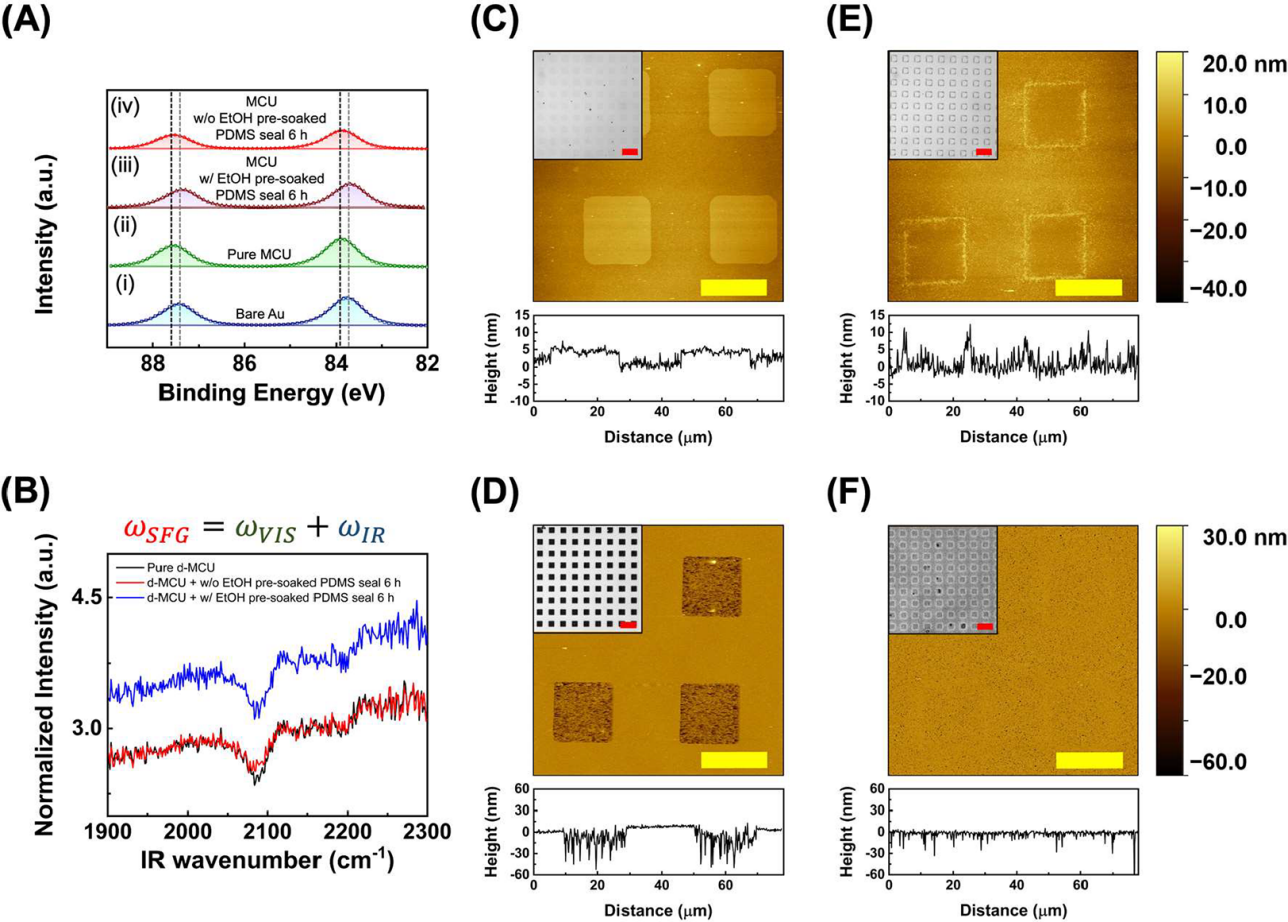
Disruption of MCU SAMs via solvent-assisted siloxane oligomer insertion. (A) Comparison of Au substrate XPS spectra (Au 4f) at various conditions: (i) bare Au, (ii) MCU-functionalized Au, (iii) MCU-functionalized Au after sealing with an ethanol-presoaked PDMS stamp for 6 h, and (iv) MCU-functionalized Au after sealing with a nonethanol-presoaked PDMS stamp for 6 h. (w/and w/o represent with and without, respectively.) The black dashed line indicates a slight shift to a higher binding energy following the formation of Au–S bond, while the gray dashed line indicates the original binding energy of bare Au. (B) SFG spectra of Au substrates covered by d-MCU under different experimental conditions with PPP polarization. The black line represents a pure d-MCU-covered Au surface, while the red and blue lines correspond to PDMS conformal sealing for 6 h using a PDMS stamp presoaked without and with ethanol, respectively. (C–F) Topographic AFM images and cross-section profiles for Au substrates fabricated by ethanol-assisted SAM disruption using PDMS stamps with protruding 20 μm square features. Upper experimental conditions: sealing for 6 h by a PDMS stamp (C) with and (E) without ethanol presoaking (30 min), respectively, before Au etching. Lower experimental conditions: sealing for 6 h by a PDMS stamp (D) with and (F) without ethanol presoaking (30 min), respectively, after Au etching. Insets of (C–F) show bright-field optical images of the corresponding Au substrates. Scale bars are 50 μm in the optical images and 20 μm in the AFM images.

A surface-specific characterization tool owing to the satisfaction of both Raman and IR selection rules in the generated signal, sum frequency generation-vibrational spectroscopy (SFG-VS), is also used to study the alkanethiol coverage and orientation on Au during the ethanol-assisted disruption process.^38–40^ SFG-VS allows for the acquisition of detailed interface molecular information and is thus employed to gain insights into this environment. To obtain information specific to the MCU thiol molecules, we substitute the terminal CH_2_ with CD_2_ (hereafter denoted as d-MCU) such that the spectra can appear in a red shift region without the interference of possible C–H signal generated from siloxane oligomers or surface contaminants. (A detailed synthetic pathway of d-MCU can be found in the Supporting Information).Figure 1B shows SFG spectra of the d-MCU-covered Au substrates with different surface treatments. For the substrate treated with ethanol-presoaked PDMS, the obvious upshift of the overall intensity compared to the original d-MCU-covered Au and the one treated with bare PDMS indicates their different SFG Au responses (nonresonant background, NR). Such a response can be an indicator of the surface thiol molecule coverage and has been discussed by previous SFG studies.^41,42^ Particularly, the NR signal decreases with rising surface coverage due to the formation of Au-thiol bonds. As shown in Figure 1B, the same NR intensity is observed for both the substrate treated with nonethanol-presoaked PDMS (red line) and the original d-MCU-covered substrate (black line), indicating their similar thiol coverage. In contrast, the increased NR signal from the sample treated with ethanol presoaked PDMS (blue line) indicates fewer Au-thiol bonds, suggesting the detachment of thiols when ethanol is involved in the process. The prominent CD_2_ symmetric stretch (CD_2_-ss) (∼2100 cm^–1^),^43^ comparably, is both orientation and quantity dependent.^44,45^ In the case of the original pure d-MCU-covered surface and the one treated with bare PDMS, their CD_2_-ss signal strength difference is below the noise level, suggesting similar molecular orientation between these two. Although reduced thiol coverage on the ethanol presoaked PDMS-treated surface would typically result in a lower CD_2_-ss signal, the unexpectedly strong CD_2_-ss signal observed suggests that signal enhancement due to disordered molecular orientation may be compensating for the lower surface coverage. Overall, the involvement of ethanol in the conformal contact sealing process not only initiates the siloxane oligomer transfer to disturb the well-ordered SAM configuration but also provides a unique environment that induces the detachment of Au-thiolates by increasing their mobility.

Based on this alkanethiol SAM disruption phenomenon, an interesting pattern transferring test that should give contradictory results to other previous observations is employed. A PDMS stamp that renders 20 μm protruding square features is preimmersed in ethanol for 30 min and then brought into contact with the MCU-modified Au substrate. After 6 h of sealing duration, the stamp is removed from the Au surface, and the Au substrate is then immersed in a wet chemical etchant containing iron­(III) nitrate and thiourea. A control experiment that uses a bare PDMS stamp without ethanol presoaking is applied for comparison, and the whole process is investigated under AFM and optical microscopy (Figure 1C–F). As demonstrated in Figure 1C, an approximately 5 nm thick PDMS residue (siloxane oligomers) accumulation at the contact region is observed when an ethanol presoaked PDMS is used. Interestingly, the areas where siloxane oligomers accumulated result in a depressed etching feature when they are subjected to a wet chemical etching process (Figure 1D). This observation is distinct from other studies that employ a protection layer on the metal but is consistent with the aforementioned spectroscopic results showing the disruption of SAMs in this conformal sealing process, which allows etchant ions to produce a pronounced metal etching effect. Comparably, trivial siloxane oligomer accumulation, except at the feature edges, is found at the contact region when a bare PDMS stamp is used during an identical test (Figure 1E). When the substrate thereafter encounters the same wet chemical etching process, no obvious etching effect occurs, as shown in Figure 1F. This suggests an ethanol-assisted effect that differentiates the interface SAM environment after the PDMS conformal sealing procedure. Since the presence of both siloxane oligomers and ethanol molecules are critical in this process, a prolonged ethanol-soaked PDMS sealing time should cause more siloxane oligomer transfer onto the Au surface and induce more severe SAM disruption (Figure S1). No significant accumulation of siloxane oligomers inside the contact region is observed after 1 h of sealing (Figure S1A), whereas an approximate 3 nm-thick layer is found after 3 h of sealing (Figure S1C). Under identical metal wet chemical etching conditions, a more obvious metal etching depth is found for 3 h (Figure S1D) than 1 h (Figure S1B) of contact sealing, consistent with the severity of the SAM disruption level.

### Roles of Siloxane Oligomers and Ethanol

The success of this SAM disruption process is expected to depend on siloxane oligomer transfer efficiency. To elucidate the behavior of siloxane oligomers deposited onto Au substrates during conformal contact, they are first extracted from PDMS and characterized by FT-IR (Figure 2A). The peaks located at 2961 cm^–1^ (Si–CH_3_, asymmetric CH_3_ stretching), 1254 cm^–1^ (Si–CH_3_, CH_3_ symmetric deformation), 1014 cm^–1^ (Si–O–Si stretching), and 790 cm^–1^ (Si–CH_3_, CH_3_ rocking) represent the characteristic features of siloxane oligomers and are well in line with the previous literature.^35,46^ Furthermore, XPS measurements are performed to analyze the transfer behavior of siloxane oligomers onto Au substrates. The Si 2p spectra can be used to identify the presence of siloxane oligomers, which should give peaks at 101.6 eV (related to the PDMS backbone Si­(−O)_2_) and 102.7 eV (assigned to Si­(−O)_3_).^47^ As shown in Figure 2B, only one component of Si 2p (at 101.6 eV) is detectable when the SAM-covered Au surface is treated with the ethanol presoaked PDMS for 1 h. Once the treatment is extended to 6 h, however, the second Si 2p peak at 102.7 eV appears, accompanied by an increase in total intensity. This indicates that increasing the conformal sealing time increases the quantity of transported siloxane oligomers, consistent with the gradual elevation observed at the PDMS-substrate contact regions in the AFM investigation (Figures S1 and 1C).

**Figure 2:**
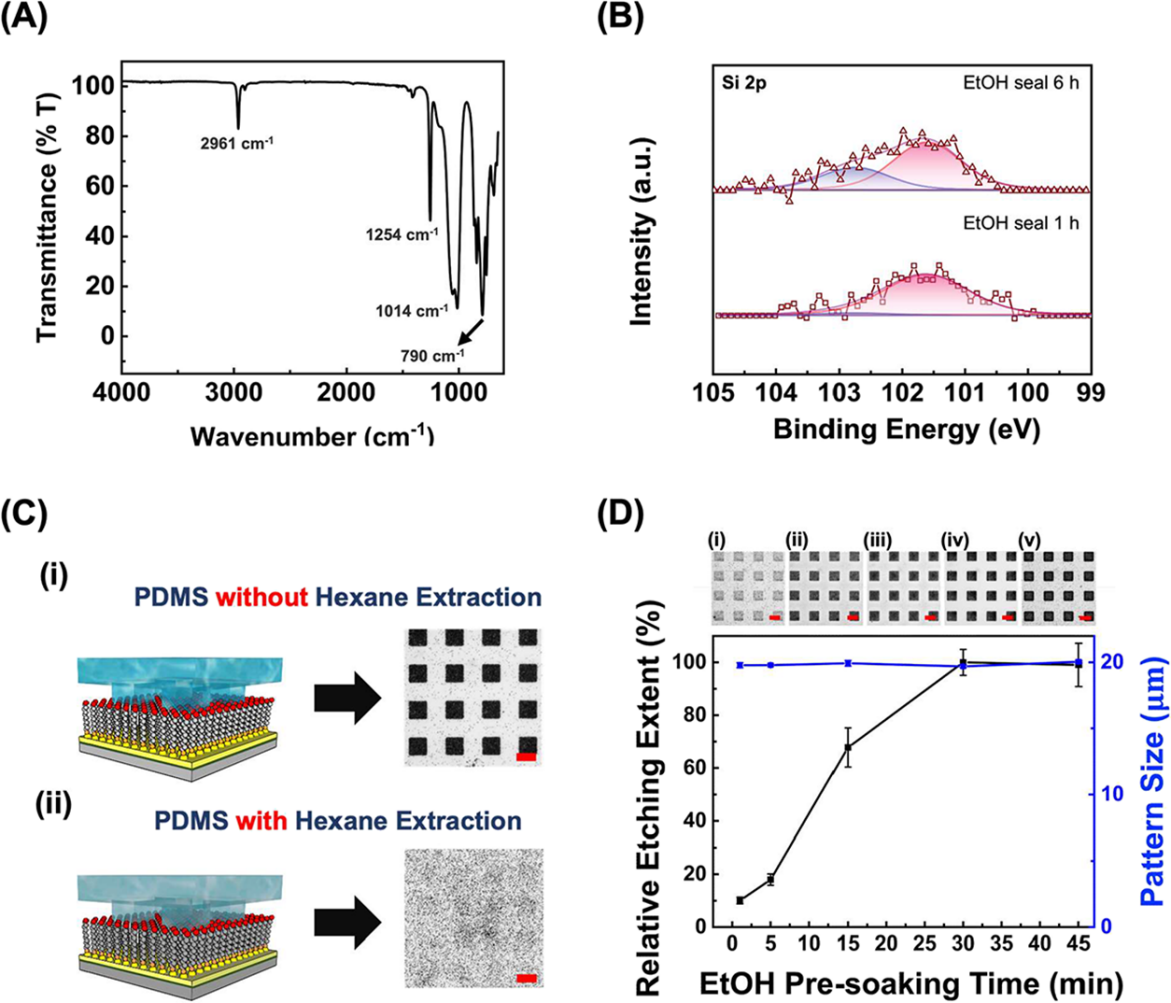
(A) FT-IR spectra of PDMS siloxane oligomer residues extracted by hexane. (B) XPS Si 2p spectra of MCU-functionalized Au substrates after conformal sealing with ethanol presoaked PDMS stamps for 1 and 6 h. (C) Optical images of MCU-covered Au substrates after PDMS conformal sealing for 6 h, followed by Au etching. When using (i) a bare PDMS stamp and (ii) a residue pre-extracted PDMS stamp. Scale bars are 20 μm. (D) The relative etching extent (black line) and the pattern size (blue line) as a function of PDMS stamp presoaking time in ethanol, with times of (i) 1, (ii) 5, (iii) 15, (iv) 30, and (v) 45 min. Scale bars are 20 μm.

A two-step mechanism is proposed for this SAM disruption process, wherein ethanol serves as both a siloxane oligomer transport medium and a mobility promoter. First, ethanol facilitates the delivery of siloxane oligomers from the PDMS stamp, allowing them to penetrate through the SAM and reach the underlying Au, thus disrupting the ordered monolayer structure. The siloxane oligomers, rich in LMW Si and O fragments, act as electron attracters.^48^ Consequently, the presence of these moieties weakens the bond energy between alkanethiol-bound Au and their adjacent Au atoms. XPS is performed to verify the electron-attracting effect from Au to oxygen-rich siloxane oligomers (Figure S2). The Au 4f_7/2_ and Au 4f_5/2_ peaks are found at 83.99 and 87.66 eV for bare Au (gray dashed line), while the transportation of siloxane oligomers on Au (black dashed line) shifts peaks to 84.14 and 87.81 eV, respectively. This binding energy upward shift confirms the electron attraction from Au to siloxane oligomers,^48^ which promotes the detachment of Au-thiolates from the bulk surface with the presence of ethanol significantly enhances the mobility of these detached species through solvation. This proposed two-step process explains the observed accumulation of PDMS residues on Au, which rather gives a depression feature after wet chemical etching, as illustrated in Figures 1C,D and S1. Standing on this point, the efficiency of SAM disruption should be highly reduced when siloxane oligomers are removed from the system, i.e., the siloxane oligomers are eliminated from the PDMS stamp. As demonstrated in Figure 2C, the MCU-covered Au delivers no etched pattern when sealed by an ethanol presoaked PDMS stamp having its siloxane oligomers extracted by hexane in advance. Compared to the result obtained using a nonhexane-treated PDMS stamp, this finding confirms the role of siloxane oligomers in disrupting SAMs. Moreover, the crucial role of ethanol’s presence in this process is illustrated in Figure 2D. A longer PDMS immersion time in ethanol means a higher quantity of solvent molecule penetration into the elastomer network. This is found to be associated with an enhanced metal etching effect on MCU-covered Au when these PDMS stamps are used to disrupt SAMs. Although ethanol causes a low PDMS swelling ratio of 1.04 and may raise feature distortion concerns,^34^ the produced pattern size is maintained when the immersion time is within 30 min. Since mobile siloxane oligomers in PDMS are consumed in the ethanol-assisted transferring process, the reusability of a PDMS stamp is also tested. As shown in Figure S3, the stamp maintains high efficacy from the first to the fourth use, with the etching depth of 44.06 ± 2.04, 43.39 ± 1.25, 43.69 ± 1.87, and 43.72 ± 1.41 nm, respectively. A pronounced decrease in etching depth by the fifth cycle (13.37 ± 1.43 nm) confirms the necessity of siloxane oligomers in this process. Relying on these material characterizations and feature transfer fidelity confirmation, a two-step SAM disruption mechanism incorporating efficient siloxane oligomer transfer and the presence of ethanol as a mobility promoter is suggested. It is also important to note that other solvents could provide similar functions and ethanol is selected here due to two primary considerations: (1) its swelling ratio approximates unity, ensuring minimal dimensional distortion and preserving pattern integrity, and (2) its widespread availability, nontoxicity, and low volatility for the convenience of operation.

### Effects of Alkanethiol Backbone Length and Terminal Group Functionality

The transfer of molecules between two substrates, e.g., in an ink-correlated printing process, depends not only on the mobility, polarity, and hydrophobicity of transferring moieties but also on the chemical properties of the destination surface.^49–51^ In the ethanol-assisted SAM disruption presented, successful delivery of siloxane oligomers from PDMS onto an alkanethiol SAM-covered Au depends on the interface environment of the corresponding substrate. For an alkanethiol SAM system, its presenting surface behavior is decided by tail group functionality, molecular arrangement, packing capacity, and interface standing orientation. Consequently, we employ different alkanethiol SAM systems, in addition to MCU described above, to investigate factors such as molecule length and terminal functionality that may influence the SAM disruption process.

In this test, a PDMS stamp with 20 μm protruding pillars presoaked in ethanol for 30 min is used, and the conformal contact sealing time toward a SAM-covered Au substrate is set at 6 h. Several alkanethiols, including 6-mercapto-1-hexanol (MCH, C_6_–OH), 16-mercapto-1-hexadecanol (MHD, C_16_–OH), 1-undecanethiol (UT, C_10_–CH_3_), and 11-mercaptoundecanoic acid (MUA, C_10_–COOH), are selected for comparison. After removal of the PDMS stamp, identical wet chemical etching is applied to the Au substrates, and the surfaces are characterized by AFM and optical microscopy. We first compare the difference between MCH (C_6_–OH) and MHD (C_16_–OH) SAMs, as shown in Figure 3A,B, which highlights the effect of molecular packing integrity on resistance to siloxane oligomer-induced SAM disruption. One would expect the longer-chain alkanethiol SAM to give a lower density of defects with a better alkane-chain ordering,^52^ which in turn provides a higher resistance toward siloxane oligomer penetration. Characterization shows a clear pattern transfer onto Au for the MCH (C_6_–OH)-covered surface, but a featureless result on the MHD (C_16_–OH)-protected one, as expected. It should also be noted that a better feature transfer is obtained for the MCU (C_11_–OH)-covered surface (Figure 1D) when compared to the MCH (C_6_–OH) obtained result under the same metal etching conditions. This is attributed to the more compact molecular alignment provided by MCU (C_11_–OH) SAM but still allowing for the penetration of siloxane oligomers in the ethanol-assisted process.

**Figure 3:**
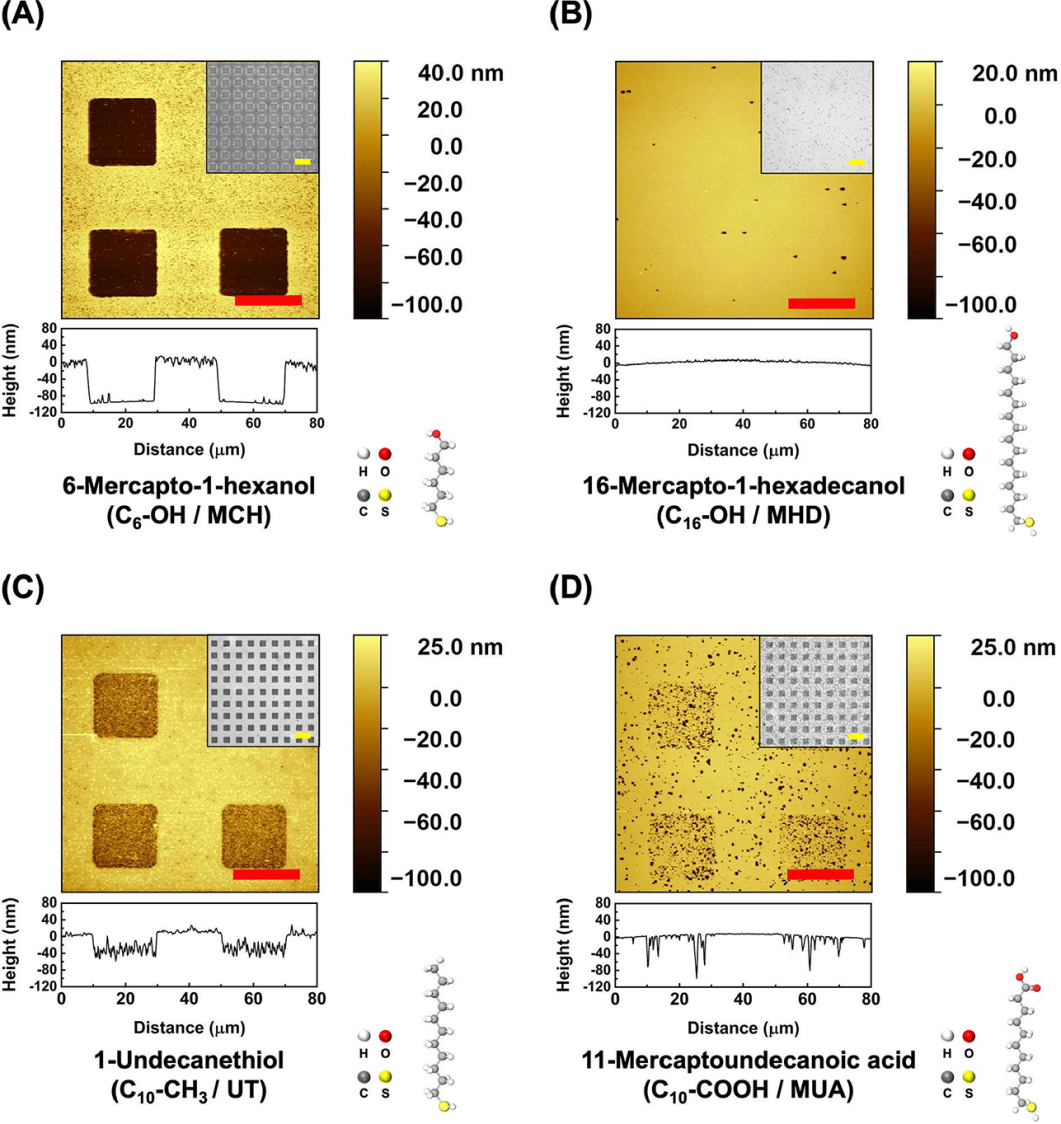
Effect of alkanethiol SAM properties toward the siloxane oligomer insertion phenomenon: (A) MCH, (B) MHD, (C) UT, and (D) MUA. Bright-field optical images (inset) and corresponding topographic AFM images with cross section profiles of substrates are shown after exposure to an ethanol presoaked PDMS stamp conformal sealing for 6 h, followed by identical Au etching treatment. Scale bars are 50 μm in the optical images and 20 μm in the AFM images.

In addition to alkane chain length, the effect of thiol molecule tail group functionality, which impacts the surface hydrophobicity, is also tested. Candidate molecules with an identical carbon number in their backbone to MCU but render a different terminal group are selected (Figure 3C,D). The 1-undecanethiol (UT, C_10_–CH_3_) with a methyl terminal group and 11-mercaptoundecanoic acid (MUA, C_10_–COOH) with a carboxyl terminal group are used to demonstrate the hydrophobicity and hydrogen bonding effect, respectively. The average etching depths are measured to be 50.17 ± 1.73, 25.25 ± 1.97, and 22.90 ± 9.93 nm for UT, MCU, and MUA molecules, respectively. Comparably, the UT molecule with a CH_3_ tail exhibits less protection against metal etching compared to MCU, evidenced by the deeper etching depth and less localized etching outside the contact area (Figure 3C). In contrast to hydroxyl-terminated MCU, the hydrophobicity of UT tends to attract mobile siloxane oligomers, which in turn promotes the penetration of these moieties and enhances the SAM disruption. Nevertheless, the interaction between UT tail groups at noncontacted areas and the gas-phase transported mobile siloxane oligomers causes a random deposition phenomenon. This counter-effect, unfortunately, diminishes the pattern contrast after the metal etching process. In comparison, the MUA molecule with a carboxylic acid (COOH) terminal group delivers a poorer feature transfer capability than MCU and UT molecules (Figure 3D). Although the average etching depth of MUA appears similar to that of MCU, a significant fluctuation in etching homogeneity with the high standard deviation in depth analysis is obtained. Even though the formation of hydrogen bonds between terminal groups of MUA hinders the penetration of siloxane oligomers, the bulkier tail group structure results in a less well-packed monolayer configuration and thus induces obvious defect sites at noncontacted areas after the metal etching process.^6,53^ In the contact sealing region, however, the insertion of siloxane oligomers still results in a comparably greater disruption effect. Overall, a heterogeneously etched surface is obtained when MUA is employed in this ethanol-assisted process.

### Selective Galvanic Metal Replacement on SAM-Disrupted Substrates

Since siloxane oligomers can be precisely transported onto a metal surface with the help of ethanol and disrupt the existing alkanethiol SAM, a subsequent interface metal redox reaction can thus be confined to this specific region. We, therefore, envision the use of this unique phenomenon for the convenient generation of substrates featuring multiple metal decoration popular in modern catalyst design, sensor assembly, and functional device fabrication.^54–56^ To achieve this goal, galvanic replacement, which spontaneously occurs between the sacrificial metal with a lower reduction potential and the target metal ion with a higher reduction potential, is employed. Galvanic replacement has been integrated with a SAM system that serves as a protection layer for the metals underneath, forming a variety of hollow and porous metallic nanostructures.^57,58^ In this work, we initiate the ethanol-assisted alkanethiol SAM disruption approach on a Pd or Pt substrate and combine it with the galvanic replacement reaction using AuCl_4_^–^ ions that can selectively reduce on the metal substrate, as demonstrated in Scheme 2.

**Scheme 2:**
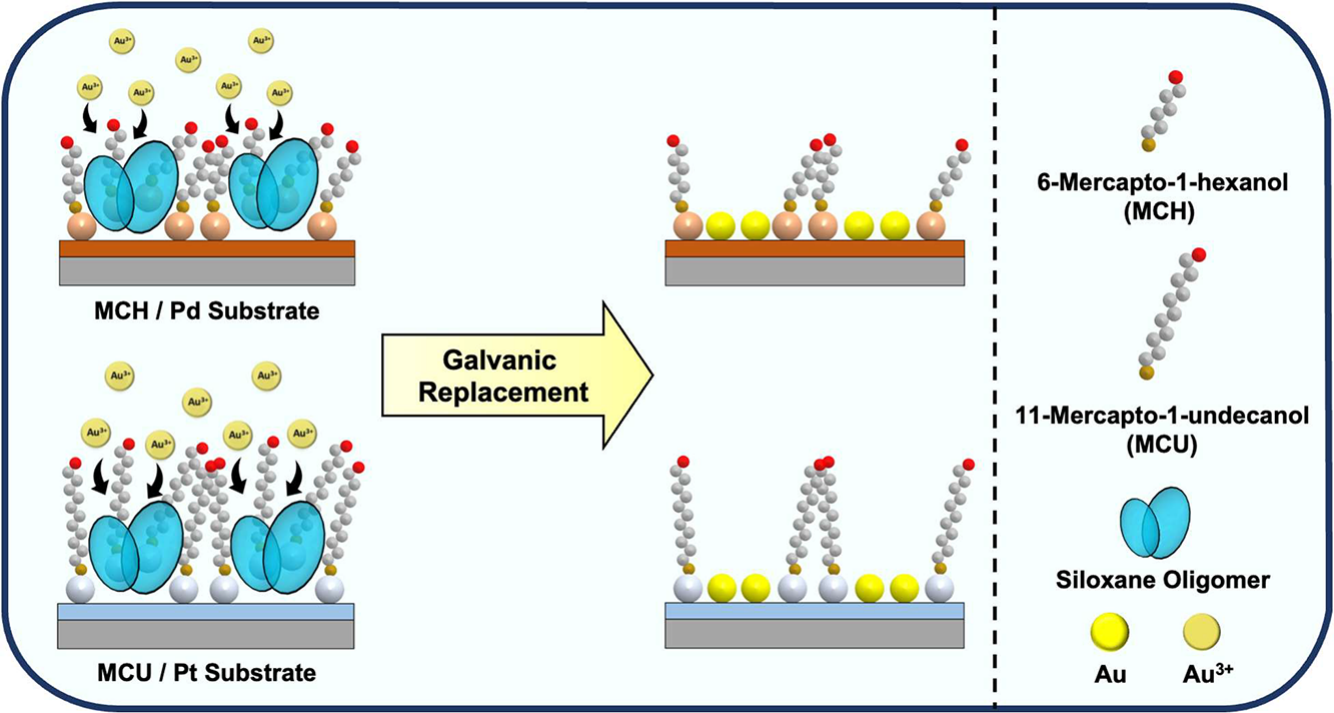
Schematic Illustration of Au Galvanic Replacement on Pd and Pt Substrates after Solvent-Assisted Alkanethiol SAM Disruption

The redox half-reactions involved can be summarized as follows:

$$\begin{array}{cc} \mathrm{Au}{{{\mathrm{Cl}}_{4}}^{-}}_{\left(\mathrm{aq}\right)}+3e^{-}\to {{\mathrm{Au}}^{0}}_{\left(s\right)}+4{{\mathrm{Cl}}^{-}}_{\left(\mathrm{aq}\right)} & \left(1.000\;V\;\mathrm{vs}\;\mathrm{SHE}\right) \end{array}$$

$$\begin{array}{cc} {}[\mathrm{Pt}{\mathrm{Cl}}_{4}{{]}^{2-}}_{\left(\mathrm{aq}\right)}+2{e^{-}}_{\left(\mathrm{aq}\right)}\to {{\mathrm{Pt}}^{0}}_{\left(s\right)}+4{{\mathrm{Cl}}^{-}}_{\left(\mathrm{aq}\right)} & \left(0.755\;V\;\mathrm{vs}\;\mathrm{SHE}\right) \end{array}$$

$$\begin{array}{cc} {}[\mathrm{Pd}{\mathrm{Cl}}_{4}{{]}^{2-}}_{\left(\mathrm{aq}\right)}+2{e^{-}}_{\left(\mathrm{aq}\right)}\to {{\mathrm{Pd}}^{0}}_{\left(s\right)}+4{{\mathrm{Cl}}^{-}}_{\left(\mathrm{aq}\right)} & \left(0.591\;V\;\mathrm{vs}\;\mathrm{SHE}\right) \end{array}$$

The preparation process includes three major steps and is briefly described as follows. First, one should notice that the arrangement of alkanethiol molecules on different metal substrates may lead to distinct tilting angles and packing capacity. Both MCH (C_6_–OH) and MCU (C_11_–OH) alkanethiols are therefore independently tested on Pd and Pt substrates to investigate their impacts on the galvanic replacement reaction (Figure S4). When the ethanol-assisted SAM disruption process is conducted on Pd substrates (Figure S4A), the use of short-chain alkanethiol MCH (C_6_–OH) exhibits an obvious Au replacement effect in the PDMS contact area in comparison to the use of long-chain MCU (C_11_–OH). This phenomenon is similar to observations in Figure 3A,B, depending upon the disruption level of SAMs. Besides, deposition of Au outside the contact region is barely seen, which is consistent with a previous study showing good metal etchant resistance when using a short-chain-length organic layer.^59^ In contrast, the use of long-chain MCU (C_11_–OH) on the Pt substrate exhibits a clearer galvanic replacement pattern than that which resulted from the use of short-chain MCH (C_6_–OH) (Figure S4B). This observation is attributed to the enhanced protection capability provided by the higher coverage and well-ordered arrangement of long-chain alkanethiols on Pt compared to shorter ones.^60^ Based on these tests, MCH (C_6_–OH) and MCU (C_11_–OH) molecules are therefore selected as the representative alkanethiols for Pd and Pt substrates, respectively, in this galvanic replacement operation. It should also be noted that the PDMS/substrate sealing time can influence the efficiency of the following galvanic replacement reaction. As shown in Figure S5, different contact sealing durations (1, 3, and 6 h) were tested on Pd and Pt substrates. An increase in sealing time correlates with a higher Au nanoparticle density within the contact area, resulting in a well-defined pattern array. This trend is consistent with the deeper etching depths obtained on Au substrates under the enhanced SAM disruption caused by longer contact sealing time.

Here, we use the MCH (C_6_–OH)/Pd combination as an example to further investigate this effective SAM disruption-induced galvanic replacement process (Figure 4). As shown in Figure 4A–C, Au nanoparticles are dispersed uniformly inside the contact-induced SAM disruption region (Figure 4B­(i)) and are barely observed outside the contact area (Figure 4B­(ii)). Conversely, a lesser quantity of Au nanoparticles is found either within or outside the contact region when a PDMS stamp without ethanol presoaking is used (Figure 4C­(i and ii)). Similar to the aforementioned SAM disruption-induced selective Au etching phenomenon (Figure 1), AuCl_4_^–^ ions can penetrate through the disrupted organic layer to oxidize the Pd metal underneath at particular regions and leave a clear Au/Pd bimetallic pattern. The selective metal replacement is also confirmed through EDS elemental analysis. The uniform distribution of Pd signal on the entire Pd substrate is obtained, whereas Au signal is only distributed within the contact-induced SAM disruption region, as shown in Figure 4D. To study the chemical state of Au decorated on a Pd substrate in this process, XPS analysis is conducted to compare the relative signal changes of both Pd and Au. As demonstrated in Figure 4E, a Pd substrate covered with MCH (C_6_–OH) presents characteristic Pd 3d_5/2_ and Pd 3d_3/2_ peaks at 335.03 and 340.29 eV, respectively. After the AuCl_4_^–^-induced galvanic replacement, the Pd 3d peaks exhibit a slight shift to higher binding energies, appearing at 335.25 and 340.51 eV. Meanwhile, the Au 4f_7/2_ (83.98 eV) and Au 4f_5/2_ (87.65 eV) peaks are only present on the Pd substrate after galvanic replacement (Figure 4E). The presence of more electronegative Au next to Pd is expected to shift the Pd signal to a higher binding energy in XPS.^61,62^ Hence, the results indicate that Au can selectively replace Pd at sites originally occupied by Pd-thiolates, once the well-packed organic layer is disrupted through ethanol-assisted siloxane oligomer insertion. Additionally, this operation can also be employed on a Pt substrate, which is premodified with MCU (C_11_–OH) molecules. As demonstrated in Figure S6A, a clear Au nanoparticle decoration boundary between the PDMS contacted (Figure S6B) and noncontacted (Figure S6C) areas is also observed, which is consistent with the Pd substrate results. Further XPS analysis (Figure 4F) in the AuPt system is also employed, where the characteristic Pt 4f peaks of a MCU-covered Pt substrate at 70.65 eV (Pt 4f_7/2_) and 73.98 eV (Pt 4f_5/2_) are monitored. After the AuCl_4_^–^-induced galvanic replacement, the Pt 4f peaks also exhibit a slight shift to higher binding energies, appearing at 70.96 and 74.29 eV. Similarly, the Au 4f peaks at 84.13 eV (Au 4f_7/2_) and 87.80 eV (Au 4f_5/2_) are only present after the galvanic replacement reaction.

**Figure 4:**
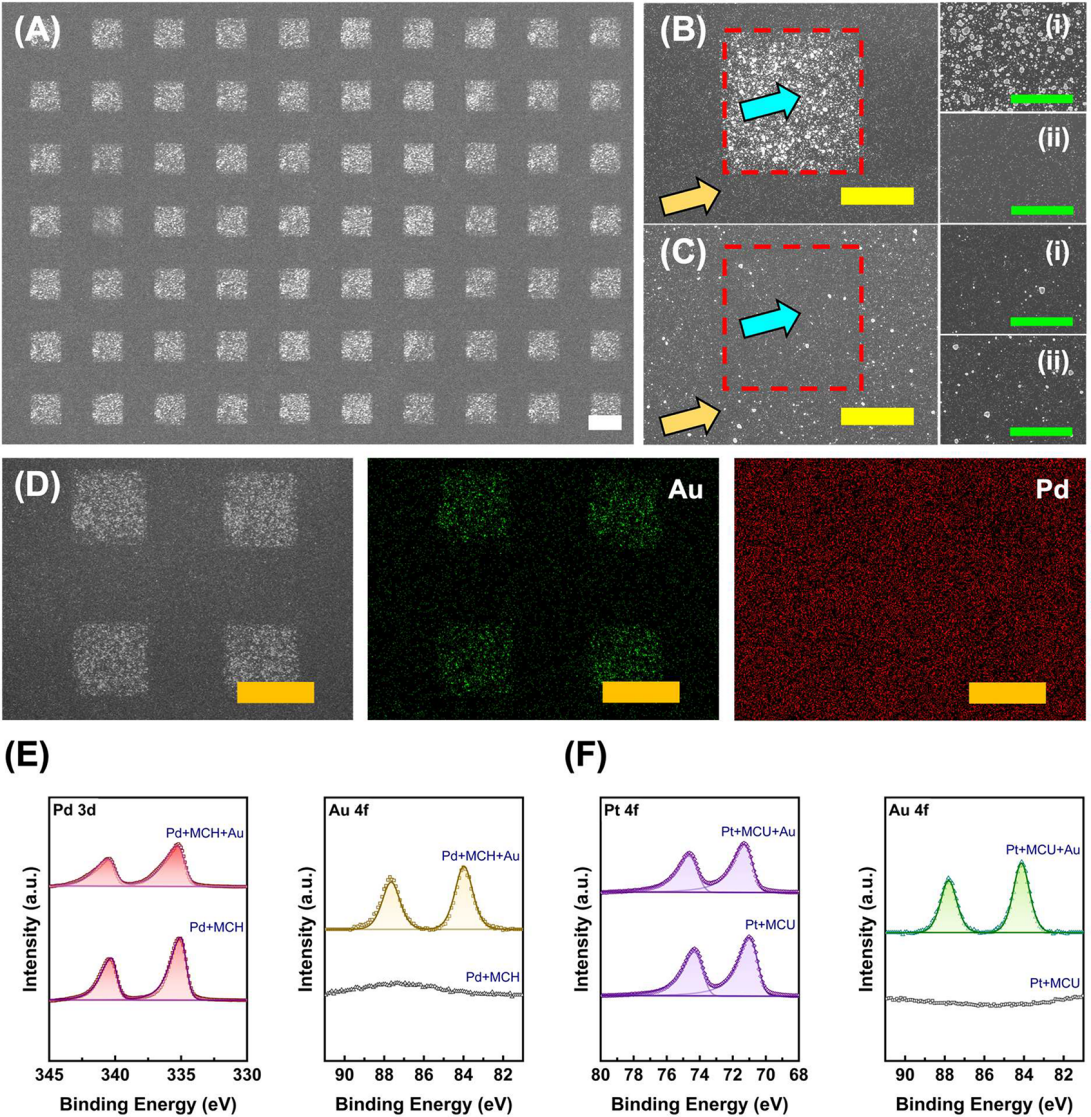
Selective Au galvanic replacement on alkanethiol SAM-disrupted metal surfaces. (A) SEM image of a MCH (C_6_OH) SAM-covered Pd substrate after ethanol-assisted SAM disruption followed by Au galvanic replacement. A PDMS stamp with 20 μm protruding square features was used for the conformal contact sealing process. The scale bar is 20 μm. (B and C) Shows SEM images comparing MCH SAM-covered Pd substrates after Au galvanic replacement when the PDMS stamp used in the SAM disruption process was pretreated (B) with and (C) without ethanol. Blue and orange arrows indicate the (i) inside and (ii) outside of the PDMS contact region (red dashed line). The scale bars for (B and C) are 10 μm and (i) and (ii) are 5 μm. (D) EDS elemental mapping of the Pd substrate after Au galvanic replacement. The scale bars are 20 μm. (E and F) XPS analysis of MCH/Pd and MCU/Pt substrates after SAM disruption-induced Au galvanic replacement.

### Metal-Enhanced Fluorescence on AuPt Bimetallic Substrates

Standing on the aforementioned successful SAM disruption-induced galvanic replacement (Figure 4), this straightforward metal decoration approach is further expanded to bimetallic systems that entail tremendous application potential. Here, we use the AuPt bimetallic system in conjunction with metal-enhanced fluorescence (MEF) effect for demonstration purposes. The MEF phenomenon occurs when fluorophores are placed in close proximity to metallic nanostructures, which can dramatically amplify fluorescence emission through plasmonic interactions.^63^ Since local electric fields can significantly influence the excitation rate and absorption cross-section of nearby fluorophores (<10 nm proximity), this enhancement is critically affected by metal nanostructure shape and size.^63–65^ Additionally, MEF is partially attributed to the scattering portion of the extinction spectrum following the radiating plasmon model, where larger metal particles with dominant scattering components provide greater MEF enhancement than smaller particles. This ultimately results in dramatically increased emission intensity when absorption and/or emission bands of fluorophores overlap with the metal scattering wavelength.

In our examination, a MCU-covered Pt substrate is first disrupted by siloxane oligomers, and the galvanic replacement is initiated to decorate Au nanoparticles above it. Since this type of fluorescence enhancement depends on spectral overlap between fluorophore excitation/emission and metal particle scattering, AuPt bimetallic substrates deposited with various dyes should provide different levels of enhancement (Figure 5A). Experimentally, these two factors can be adjusted through the metal nanoparticle growing process and the alkanethiol monolayer disruption extent. To achieve optimal metal nanoparticle growth, we systematically examined the relationship between HAuCl_4_ precursor concentration and its corresponding MEF enhancement effect, utilizing Rhodamine 6G (R6G) as a model fluorophore (Figure S7). As evidenced in Figure S7A, large but irregularly shaped nanoparticles with low surface density are observed when the 1 mM HAuCl_4_ condition was applied. This configuration proves suboptimal for MEF due to insufficient enhancement environment generation. Conversely, the use of 0.1 mM HAuCl_4_ (Figure S7B) yields the most favorable condition, producing high-density nanoparticles of appropriate size that maximize hot spot formation and electric field enhancement to give the strongest MEF effect. At the lowest concentration of 0.01 mM (Figure S7C), while particle uniformity improves, the combination of small particle size and inadequate density results in negligible MEF effects. Based on these findings, 0.1 mM HAuCl_4_ was selected as the optimal particle growing condition for subsequent MEF investigations with different fluorophores. Beyond precursor concentration, the PDMS sealing duration, which decides the alkanethiol monolayer disruption, emerges as the other critical parameter, as demonstrated in Figure S8. It should be noted that the extended PDMS sealing time promotes the disruption level of alkanethiol SAM, which creates more reactive sites for subsequent galvanic replacement. Progressively increased Au nanoparticle size and density within the disrupted regions are therefore observed. Relying on this effect, we investigated the relationship between MEF enhancement and PDMS sealing time by employing three different fluorescence dyes: fluorescein isothiocyanate (FITC), rhodamine 6G (R6G), and cyanine5.5 amine (Cy5.5). As shown in Figure 5B, these dyes exhibit distinct fluorescence emission trends depending on their coherent interaction with the bimetallic platform. Cy5.5 demonstrates continuous fluorescence enhancement with increasing sealing time, while R6G exhibits an initial enhancement followed by a modest decline at extended durations. In contrast, FITC consistently shows minimal fluorescence enhancement across all tested sealing times. These differential responses can be attributed to overlaps between fluorophore excitation and emission wavelengths with the nanoparticle localized surface plasmon resonance (LSPR) band, which is correlated to the evolution of Au nanoparticle morphology on bimetallic substrates. As PDMS sealing time increases, larger and more anisotropic Au nanoparticles form, resulting in red-shifted LSPR bands.^66^ Assuming spherical geometry and utilizing average particle sizes for each condition, the increasing particle size broadens the scattering peaks and shifts them toward longer wavelengths with enhanced intensity (Figure S9). As compared in Figure 5C, the particle scattering cross-section spectrum exhibits optimal overlap with employed fluorophore excitation and emission wavelengths when ∼259 nm Au particles are generated (6 h sealing) on Pt substrates. The spectral overlap analysis reveals that the superior overlap of Cy5.5′s spectra compared to R6G and FITC indeed corresponds to its best MEF performance.

**Figure 5:**
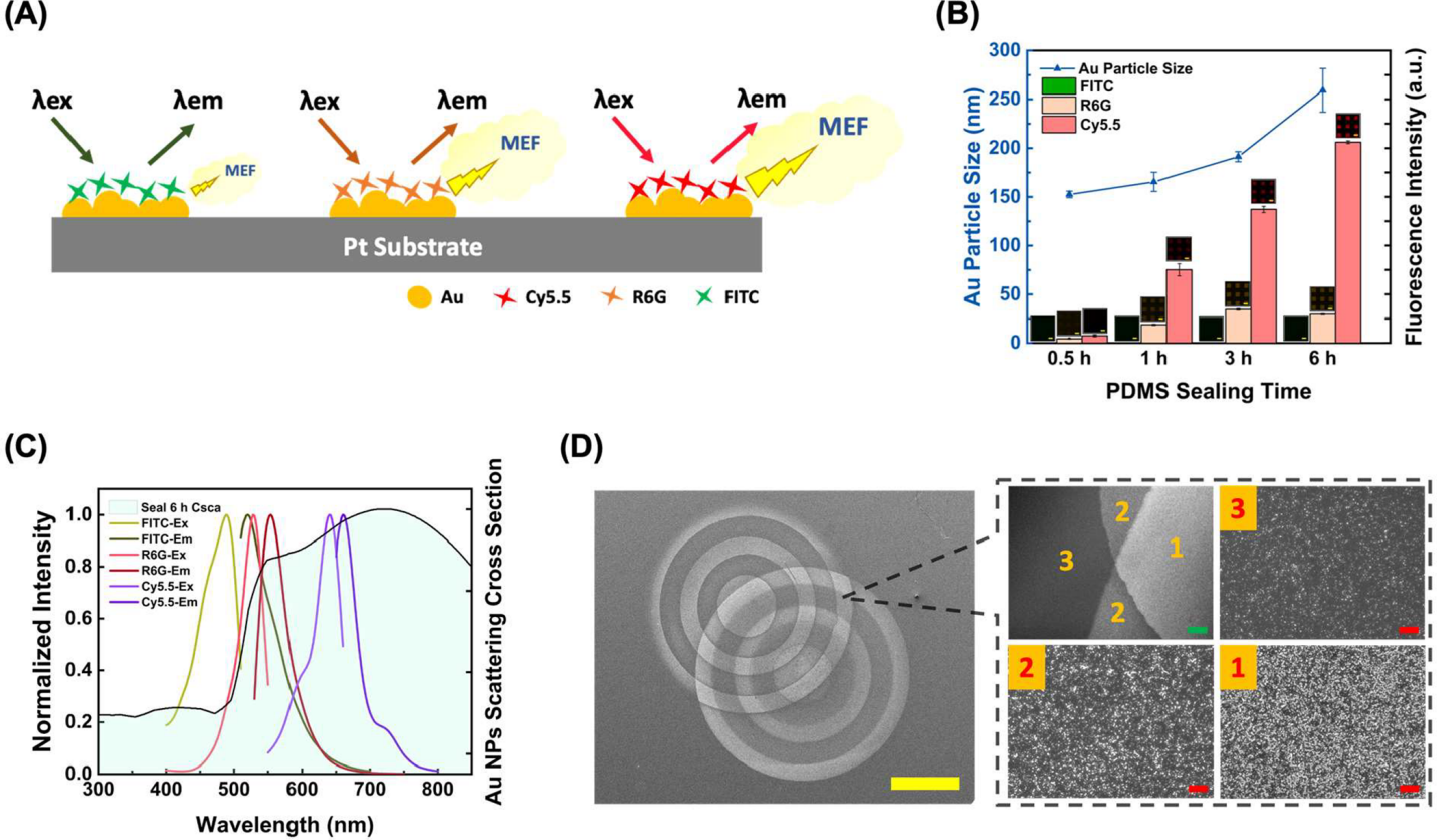
Metal-enhanced fluorescence enhancement effects induced by integrating AuPt bimetallic substrates with various dyes. (A) Schematic illustration of different levels of MEF effect when using distinct fluorescent dyes. (B) Au nanoparticle size-dependent fluorescence emission of AuPt bimetallic substrates prepared by different PDMS sealing times. Fluorescence dyes used: FITC (excitation: 495 nm, emission: 519 nm), R6G (excitation: 555 nm, emission: 569 nm), and Cy5.5 (excitation: 651 nm, emission: 670 nm). (C) Scattering cross-section of Au nanoparticles (sealing for 6 h) synthesized on Pt substrates, overlaid with the excitation and emission spectra of each fluorescent dye. (D) SEM images of overlaid concentric ring features fabricated by PDMS double sealing on MCU SAM-covered Pt with subsequent Au galvanic replacement. The right boxed area shows a magnified view of the concentric ring features. (Region 1: total sealing for 6 h; Region 2: total sealing for 3 h; Region 3: without sealing.) Scale bars: yellow = 500 μm; green = 20 μm; red = 1 μm.

It is important to note that this alkanethiol SAM disruption is an accumulative process and can thus be repeated on the same substrate to create multiplexed SAM-disrupted regions. For demonstration, concentric circle patterns are designed to create overlapping structures through a two-step sealing process, with each step lasting 3 h. This feature enables the generation of continuous, intersecting areas where alkanethiol SAMs are disrupted to varying degrees, thereby facilitating distinct galvanic replacement. As evidenced in the SEM images shown in Figure 5D, Au nanoparticles with various dimensions and density are spatially addressed on the Pt substrate. Features with two distinct concentric rings are formed, where double-sealed regions (region 1) carry denser and larger Au nanoparticles compared to single-sealed (region 2) or unsealed regions (region 3). This is consistent with the SAM disruption extent that provides active sites for subsequent metal nucleation and growth. Taking advantage of the accumulative property in this operation, we envision the use of a multiplexed, diverse density Au nanoparticle-decorated array to combine with the previously described MEF phenomenon as a unique, intrinsic internal standard-equipped analytical platform. In this design (Figure 6A), a PDMS stamp featuring a 3 μm wide linear shape pattern is employed in a two-step sequential sealing (an initial 2 h sealing followed by a secondary 4 h sealing) to provide a spatially addressed multilevel SAM-disrupted substrate. This surface is thereafter utilized to create distinct regions carrying diverse Au nanoparticle densities, as evidenced in Figure 6C. A blue water-soluble pigment, phycocyanin (with characteristic excitation and emission at 622 and 646 nm, respectively, Figure 6B), is then applied onto this substrate as a model fluorophore for MEF tests. As demonstrated in Figure 6C inset, different fluorescence responses from distinct regions resemble the distribution of Au nanoparticles on the same substrate. Highly magnified SEM images (Figure 6C, right panel) clearly reveal four distinct zones: Region *a* (6 h total sealing time), Region *b* (4 h sealing), Region *c* (2 h sealing), and Region *d* (noncontacted control region). The consistency of differential fluorescence enhancements observed across *a*–*d* regions with surface Au nanoparticle distribution confirms the capability of this approach to guide MEF effects on the same substrate. Based on this, an analytical platform that can facilitate signal normalization with enhanced analysis robustness through substrate-equipped intrinsic internal standards is designed. To validate this concept, two types of analytical approaches are conducted and compared simultaneously. Similar to conventional analytical methods, calibration curves are built upon fluorescence intensity (gray scale value analyzed by image J software) versus phycocyanin concentration for *a*–*c* regions on the same substrate. As demonstrated in Figure 6D­(i), the *R* ^2^ values of linear curves for region *a*, *b*, and *c* are calculated to be 0.946, 0.931, and 0.915, respectively, which represent the substrate’s capability for analyte detection. Comparably, a platform-equipped internal standard strategy through utilizing Region *c* as a reference is further implemented. This approach leverages the ratiometric measurements between target and reference regions, which effectively normalizes signal background fluctuation and interference in various experimental conditions since all the signals are collected from the identical substrate. Furthermore, the built of calibration curve and real sample detection are realized on the same chip simultaneously, which effectively eliminates the dependency on chip-to-chip absolute reproducibility. As shown in Figure 6D­(ii), the *R* ^2^ values of linear curves in the fluorescence intensity ratio (target region/region *c*) versus analyte concentration plot are further improved to be 0.999 (for *a*/*c*) and 0.998 (for *b*/*c*), respectively. This platform-equipped internal standard analytical method significantly improves the linearity of calibration curves, thus improving the accuracy and reproducibility of unknown sample quantification on the platform. It is important to note that the superior analytical performance described above relies on creating multiplexed signaling regions on an identical substrate, which cannot be supported by conducting replicate experiments to collect signals from different sources.

**Figure 6:**
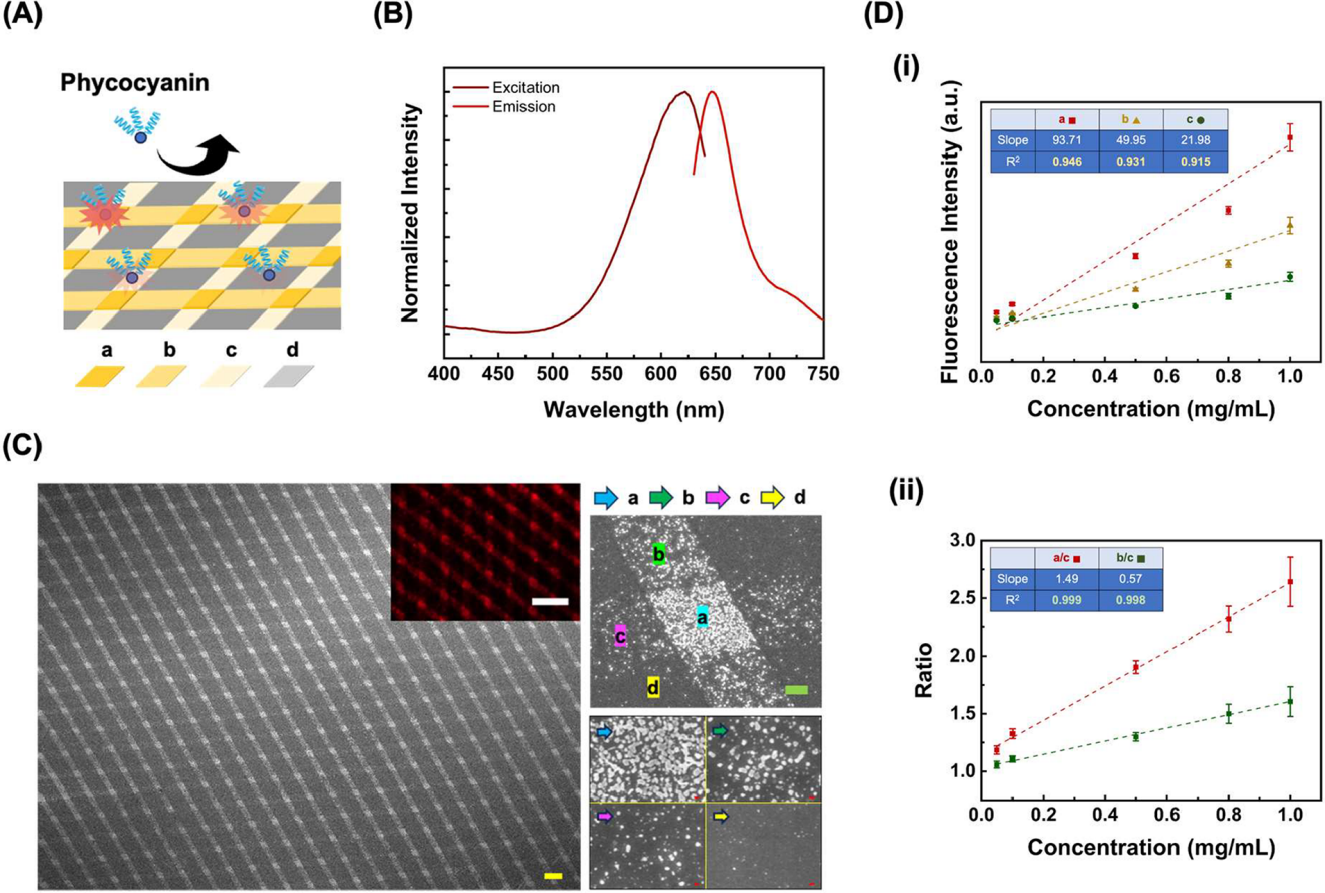
MEF-dependent intrinsic internal standard-equipped analytical platform. (A) Schematic illustration of phycocyanin detection on a multiplexed AuPt substrate. (B) Excitation/emission spectra of phycocyanin. (C) Left: A large-scale SEM image of the prepared multiplexed AuPt substrate. The inset shows its corresponding fluorescence image under 651 nm of excitation. Right: A magnified view of the intersected 3 μm wide line features. (Region a: total sealing for 6 h; Region b: total sealing for 4 h; Region c: total sealing for 2 h; Region d: without sealing.) Scale bars: yellow = 10 μm; white = 20 μm; green = 1 μm; red = 100 nm. (D) (i): Calibration curves showing the relationship between fluorescence intensity and phycocyanin concentration. (ii): Calibration curves showing the relationship between fluorescence intensity ratio and phycocyanin concentration using Region c as the internal standard.

To examine the detection accuracy of this unique method, recovery tests on real samples are conducted. A certain quantity of phycocyanin is spiked into real sample matrices, with each level measured in triplicate trials (*N* = 3). This experiment evaluates whether the described platform-equipped internal standard approach can accurately quantify analytes in the presence of complex matrices and potential interferents. The average recoveries obtained for tap water (>91%) and green tea (>95%) samples demonstrate the satisfactory applicability of this approach for reliable phycocyanin determination in environmental and food-related samples (Table 1). These findings emphasize the potential of this platform for other biosensing, bioimaging, and advanced information analysis applications.

**Table 1:** Recovery Tests Using Tap Water and Green Tea as the Real Sample Matrix, Which Are Spiked with 0.2 and 0.5 mg/mL of Phycocyanin, Respectively (*N* = 3)^a^

sample	added (mg/mL)	measured (mg/mL)	RSD	recovery (%)
tap water	0.2	0.182	0.015	91.22
green tea	0.5	0.478	0.040	95.69

a The measured concentration is calculated by substituting the fluorescence intensity ratio (region a/region c) into the concentration calibration curve.

## Conclusions

An unconventional ethanol-assisted alkanethiol SAM disruption approach is introduced. Different from previous feature fabrication or surface patterning reports, the notorious siloxane oligomers in elastomeric polymer, i.e., PDMS, are utilized as a practical tool in lieu of common surface contaminants. Movements of siloxane oligomers from polymerized elastomers are controlled through the assistance of pre-entrapped ethanol, which can be transferred selectively onto a substrate during a conformal contact sealing process. These mobile moieties disrupt preformed alkanethiol SAMs on Au substrates, and lead to the detachment of interface Au-thiolates. The presence of this unusual phenomenon originates from a unique interface mobile environment that supports controllable movement of ethanol and siloxane oligomer molecules at restricted polymer/substrate contact regions. Spectroscopic investigation of this interface environment via SFG-VS and XPS analysis points to an oxygen-rich environment that attracts electrons from surrounding Au atoms, induced by mobile siloxane oligomers. This weakens the bond energy between Au-thiolates and the surrounding Au atoms, thus promoting their detachment from the substrate. Compared to most siloxane oligomer transfer studies that utilize the siloxane oligomers as a protection layer, the generated protruding feature on the surface allows the penetration of oxidizing ions in aqueous solution and results in inverse feature creation on Au substrates. Prolonged ethanol presoaked PDMS contact duration increases siloxane oligomer accumulation and distinct feature transfer, while the absence of these moieties eliminates this SAM disruption behavior. Importantly, the alkanethiol chain length and terminal functional group properties both severely affect the extent of SAM disruption, depending upon the ease of interface environment-allowed siloxane oligomer transportation. These observations further confirm the function of ethanol and siloxane oligomers in this system, which can be carefully controlled through precise molecular positioning. Taking advantage of this confinable SAM disruption process, versatile alkanethiol systems on Pd and Pt substrates enable selective Au nanoparticle formation at SAM-disrupted regions through the galvanic replacement reaction. These spatially addressed bimetallic substrates support metal-enhanced fluorescence, which is Au particle size and fluorophore excitation/emission property dependent. By multiplexing galvanic replacements on the same substrate, distinct levels of fluorescence enhancements are achieved on the same surface. This provides the opportunity to build multiple internal standards on the same substrate that realizes the minimization of errors in imaging analysis through ratiometric treatments. Several examinations of dye solutions and real samples with recovery tests confirm the feasibility of this design, enabling a platform that delivers a significant reduction in analytical errors. We anticipate that this type of substrate can serve as a practical analytical tool, offering flexibility for a range of bioimaging applications and enabling precise data analysis.

## Experimental Section

### Materials and Chemicals

6-Mercapto-1-hexanol (MCH), 11-Mercaptoundecanol (MCU), 1-undecanethiol (UT), Fluorescein 5 (6)-isothiocyanate (FITC), Rhodamine 6G (R6G), and hexamethyldisilazane (HMDS) were purchased from Sigma-Aldrich (St. Louis, MO, USA). 16-Mercapto-1-hexadecanol (MHD) was purchased from Matrix Scientific (Columbia, SC, USA). Anhydrous ethanol, acetone (99.5%), hexane, and isopropanol (99.9%) were purchased from Echo Chemical (Taipei, Taiwan). Hydrogen peroxide (H_2_O_2_), iron­(III) nitrate nonahydrate (Fe­(NO_3_)_3_·9H_2_O), and thiourea (CS­(NH_2_)_2_) were purchased from SHOWA (Tokyo, Japan). Sulfuric acid (H_2_SO_4_) was purchased from Fluka-Honeywell (Charlotte, NC, USA). Hydrogen tetrachloroaurate­(III) trihydrate (HAuCl_4_·3H_2_O) was purchased from Alfa Aesar (Lancashire, UK). Phycocyanin from *Spirulina* was purchased from Tokyo Chemical Industry (Tokyo, Japan). Silicon wafers were provided by Mustec Corp. (Hsinchu, Taiwan). Cyanine5.5 amine (Cy5.5) was obtained from Lumiprobe Corp. (Maryland, USA). SYLGARD 184 silicone elastomer base and curing agent were purchased from Dow Corning Corp. (Midland, MI, USA). Positive photoresist AZ6112 was purchased from AZ Electronic Materials Taiwan Co., Ltd. (Taipei, Taiwan). T238 developer was purchased from Control Chemitech Inc. (Taoyuan, Taiwan). Ultrapure water (>18.2 MΩ·cm) generated from an ELGA PURELAB classic system (Taipei, Taiwan) was used throughout all experiments.

### Instruments

Atomic Force Microscopy (AFM) images were obtained with a Bruker Dimension Fastscan instrument (Bruker Nano Surfaces, Hsinchu, Taiwan). Optical images were collected by a Zeiss epifluorescence microscope (Axio Imager. M2, Carl Zeiss Microscopy, Jena, Germany). Sum frequency generation spectroscopy (SFG) spectra were obtained by an in-house sum frequency vibrational spectroscopic setup using a Ti:sapphire laser system (Astrella, Coherent). The detailed SFG setup can be found in our previous work.^67^ But briefly, all beams were set to be p-polarized to ensure an adequate signal-to-noise ratio in SFG measurements. The exposure time for each presented spectrum was 60 s, with at least two different spatial locations and normalized against z-cut quartz. X-ray photoelectron spectroscopy (XPS) measurements were performed with a PHI Quantes spectrometer (ULVAC-PHI, Inc., Japan) using a monochromatic Al Kα (1486.6 eV) light source. A total resolution of 0.1 eV and a beam size of 200 μm were maintained, utilizing a pass energy of 55 eV. Binding energies were calibrated by setting C 1s to be 284.5 eV. Scanning electron microscopy (SEM) images were obtained by a JSM-6700F field-emission scanning electron microscope (JEOL Ltd., Tokyo, Japan). Fourier-transform infrared (FTIR) spectra were obtained from a JASCO FTIR-4600 spectrometer with an attenuated total reflection accessory on an integrated ZnSe prism.

### Synthesis of d-MCU (**4-1**)

The overall synthetic routes for compounds are depicted in Scheme S1 and full synthetic details and characterization for d-MCU and its intermediates are provided in the Supporting Information.

#### Methyl 11-Bromoundecanoate (**2-1**)^68^

To a solution of 11-bromoundecanoic acid (1.53 g, 5.77 mmol, 1.0 equiv) in anhydrous MeOH (19.0 mL) was added acetyl chloride (2.0 mL, 28.84 mmol, 5.0 equiv) under an ice bath. After the mixture was stirred for 3 h at room temperature, the volatiles were removed under reduced pressure. The residue was resuspended in diethyl ether, and the organic layer was washed with saturated NaHCO_3 (aq)_ three times. The combined organic layer was washed with brine, dried over Na_2_SO_4_, filtered off the solid, and concentrated to obtain **2-1** as a yellow oil at 1.48 g in 92% yield. *R*_f_ = 0.4 (hexane/EtOAc = 9/1). ^1^H NMR (400 MHz, CDCl_3_): δ 3.67 (s, 3H), 3.40 (t, *J* = 6.9 Hz, 2H), 2.30 (t, *J* = 7.5 Hz, 2H), 1.85 (m, 2H), 1.62 (m, 2H), 1.42 (m, 2H), 1.32–1.29 (m, 10H).

#### Methyl 11-(Acetylthio)­undecanoate (**2-2**)^69^

To a solution of **2-1** (2.76 g, 9.90 mmol, 1.0 equiv) in anhydrous DMF (9.0 mL), potassium thioacetate (2.71 g, 23.8 mmol, 2.4 equiv) was separately added in anhydrous DMF (19.8 mL) dropwise under an ice bath. The mixture was stirred for 1 h at room temperature under argon atmosphere, diluted with water, and extracted with diethyl ether (20.0 mL) five times. Then the combined organic layer was washed with brine, dried over Na_2_SO_4_, filtered off the solid, and concentrated under vacuum. Purification by flash column was carried out with a solvent composition of EtOAc/hexane = 0/100 to 5/100 to obtain the **2-2** as a yellow solid (2.40 g, 88% yield). *R*_f_ = 0.38 (hexane/EtOAc = 9/1). ^1^H NMR (400 MHz, CDCl_3_): δ 3.66 (s, 3H), 2.85 (t, *J* = 7.4 Hz, 2H), 2.31 (s, 3H), 2.29 (t, *J* = 7.4 Hz, 2H), 2.32–2.28 (m, 5H), 1.63–1.52 (m, 4H), 1.36–1.27 (m, 12H). IR (neat): 2927, 2854, 2357, 1740, 1693, 1435, 1354, 1247, 1197, 1171, 1134, 1110, 952 cm^–1^. HRMS (ESI-TOF) calculated for C_14_H_26_NaO_3_S (M + Na)^+^ 297.1495, found 297.1484.

#### 11-Mercaptoundecan-1-ol (**1-2**)

To a solution of LiAlH_4_ (40 mg, 1.07 mmol, 3.0 equiv) in anhydrous THF (1.0 mL) was added the solution of **2-2** (98 mg, 0.36 mmol, 1.0 equiv) in anhydrous THF (1.0 mL) dropwise under an ice bath, and the mixture reacted at room temperature for 30 min under argon atmosphere. The mixture was worked up with saturated NH_4_Cl_(aq)_ (2.5 mL) and saturated Na_2_SO_4(aq)_ (2.5 mL) under an ice bath, and then stirred for 10 min at room temperature. Then the mixture was diluted with EtOAc (5.0 mL) and stirred for another 10 min. The solid was filtered off through Celite and washed with EtOAc (2.0 mL) three times. The filtrate was combined and washed with saturated NH_4_Cl _(aq)_ (30.0 mL) and brine (30.0 mL) three times. The organic layer was dried over Na_2_SO_4_, filtered off the solid, and concentrated under vacuum. Finally, the residue was taken up with methanol and recrystallized from water. The solution was centrifuged and the liquid layer was removed to obtain **1-2** as a white solid (54 mg, 74% yield). *R*_f_ = 0.3 (hexane/EtOAc = 7/3). ^1^H NMR (400 MHz, CDCl_3_): δ 3.64 (t, *J* = 6.6 Hz, 2H), 2.52 (q, *J* = 7.4 Hz, 2H), 1.64–1.53 (m, 4H), 1.37–1.28 (m, 14H). ^13^C NMR (100 MHz, CDCl_3_): δ 63.2 (CH_2_), 34.2 (CH_2_), 32.9 (CH_2_), 29.7 (CH_2_), 29.6 (2 CH_2_), 29.5 (CH_2_), 29.2 (CH_2_), 28.5 (CH_2_), 25.8 (CH_2_), 24.8 (CH_2_). IR (neat): 3349, 2924, 2853, 1465, 1056, 718 cm^–1^. HRMS (ESI-TOF) calculated for C_11_H_23_OS (M-H)^−^ 203.1470, found 203.1468.

#### 11-Mercaptoundecan-1,1-d2–1-ol (**4-1**)

To a solution of LiAlD_4_ (215 mg, 5.13 mmol, 3.0 equiv) in anhydrous THF (2.0 mL) was added the solution of **2-2** (469 mg, 1.71 mmol, 1.0 equiv) in anhydrous THF (2.0 mL) dropwise under an ice bath, and the mixture was reacted at room temperature for 5 min under argon atmosphere. The mixture was worked up with saturated NH_4_Cl _(aq)_ (10.0 mL) and saturated Na_2_SO_4 (aq)_ (10.0 mL) under an ice bath, and the mixture was stirred for 10 min at room temperature. Then the mixture was diluted with EtOAc (20.0 mL) and stirred for another 10 min. The solid was filtered off through Celite and washed with EtOAc (10.0 mL) three times. The filtrate was collected and extracted with saturated NH_4_Cl _(aq)_ (30.0 mL) and brine (30.0 mL) three times. The organic layer was dried over Na_2_SO_4_, filtered off the solid, and concentrated under vacuum. Finally, the residue was taken up with methanol and recrystallized from water. The solution was centrifuged and the liquid layer was removed to obtain **4-1** as a white solid (268 mg, 76% yield). *R*_f_ = 0.3 (hexane/EtOAc = 7/3). ^1^H NMR (400 MHz, CDCl_3_): δ 2.52 (q, *J* = 7.3 Hz, 2H), 1.64–1.53 (m, 4H), 1.32–1.28 (m, 14H). ^13^C NMR (100 MHz, CDCl_3_): δ 62.4 (quin, *J* = 21.6 Hz, CD_2_), 34.2 (CH_2_), 32.7 (CH_2_), 29.7 (CH_2_), 29.6 (2 CH_2_), 29.5 (CH_2_), 29.2 (CH_2_), 28.5 (CH_2_), 25.8 (CH_2_), 24.8 (CH_2_). IR (neat): 3373, 2924, 2853, 1460, 1243, 958 cm^–1^. HRMS (ESI-TOF) calculated for C_11_H_22_^2^H_2_OS (M)^+^ 206.1673, found 206.1684.

### Alkanethiol Self-Assembled Monolayer Preparation

Silicon substrates with a 5 nm-thick Cr adhesion layer and a 100 nm-thick Au layer were prepared by thermal evaporation. The Au substrates were first cleaned with piranha solution (3:1 H_2_SO_4_/H_2_O_2_) for 30 min and then washed with DI water (18.2 MΩ·cm) several times. Afterward, the cleaned Au substrates were incubated in 1 mM alkanethiol ethanolic solution overnight. After removal from the thiol solution, the Au substrates were rinsed thoroughly with ethanol to remove physisorbed thiol molecules and then blown dry with nitrogen gas.

### Patterned Master Mold Fabrication

The patterned master mold was acquired through the standard photolithography method. A 2-in. silicon wafer was rinsed with isopropanol and baked at 120 °C for 15 min and placed into a HMDS vapor chamber upside down for another 15 min. An adhesion layer of HMDS on the silicon wafer was obtained owing to the chemical vapor deposition process. Positive photoresist AZ6112 was coated onto the silicon wafers via spin-coating with the first 10 s rotating at 800 rpm and another 50 s rotating at 1200 rpm. Afterward, the wafers were baked at 120 °C for 5 min and exposed to UV light (30 mW/cm^2^ for 0.8 s) along with the patterned photomask. Then, T238 developer was utilized to wash away AZ6112 in the area with UV exposure and a final bake at 120 °C for 10 min completed the master mold fabrication.

### Preparation of PDMS Stamps

The PDMS stamps were prepared by thoroughly mixing the SYLGARD 184 silicone elastomer base and curing agent with a mass ratio of 10:1, followed by degassing the mixture in a vacuum desiccator to remove air bubbles. Subsequently, the PDMS mixture was cast onto a HMDS-coated master mold containing a 20 μm × 20 μm square hole array, as described above, and cured at 80 °C for 2 h. Finally, the solidified PDMS was separated from the master mold and cut into 1 cm × 1 cm pieces for further use.

### Ethanol-Assisted SAM Disruption

PDMS stamps were immersed in anhydrous ethanol for a certain period to allow ethanol permeation into the porous structure of PDMS. Subsequently, the PDMS stamps were removed from the ethanol solution, dried with nitrogen gas, and directly sealed onto the SAM-functionalized Au substrate. After different sealing durations, the PDMS stamps were carefully peeled off the Au substrates. The SAM-disrupted surfaces were then ready for further treatment.

### Wet Chemical Etching of Au

An oxidation–reduction process at the metal–solution interface, using iron­(III) nitrate as an oxidant and thiourea as an effective Au ligand, was applied to initiate the Au wet etching.^70,71^ In this operation, SAM-disrupted Au substrates were immersed in an etching solution consisting of 40 mM thiourea and 60 mM iron­(III) nitrate for 30 min. The resultant etched surfaces were then washed with copious amounts of DI water, blown dry with nitrogen gas, and then ready for further characterization. Relative etching extent in Figure 2D obtained from AFM image was quantified by subtracting the height within the patterned area (*N* = 6) from the background height (outside the patterned area).

### Siloxane Oligomer Extraction from Solidified PDMS

To extract siloxane oligomers from solidified PDMS, the PDMS stamp was immersed in 40 mL of hexane at room temperature and kept under stirring for 3 h, with the hexane solution replaced by fresh solvent every hour. Afterward, the PDMS stamp was removed from hexane and transferred to 40 mL of ethanol at room temperature. After another 3 h of stirring and replacing the solvent every hour, the PDMS stamp was dried in a vacuum oven at 75 °C for 2 h and was ready for further use.^72^

### Galvanic Replacement on SAM-Disrupted Metal Substrates

To generate SAM-disrupted metal substrates for galvanic replacements, PDMS stamps (rendering square-shaped pillars) preimmersed in anhydrous ethanol for 30 min were used. These PDMS stamps were sealed onto the SAM-functionalized Pd or Pt substrates for a certain period of time and then carefully peeled off. The produced SAM-disrupted metal substrates were thereafter immersed in 1 mM HAuCl_4_ aqueous solution at 30 °C overnight to complete the galvanic replacement process.

### Metal-Enhanced Fluorescence Measurements

Patterned AuPt bimetallic substrates were immersed in solutions containing different fluorescent dyes (each at a concentration of 10^–6^ M) for 6 h. A fluorescence microscope equipped with dye-specific filters was used to observe the metal-enhanced fluorescence effect for each dye. Fluorescence intensity obtained from ImageJ software was quantified by subtracting the background signal (outside the patterned area) from the intensity within the patterned area (*N* = 5). The resulting values were then normalized and analyzed. Mie scattering cross-section calculations for the synthesized Au nanoparticles in air were performed using freeware MiePlot v4.6 software.

### Phycocyanin Detection Platform

The intersected line-shape-patterned AuPt bimetallic substrates were fabricated through a two-step sequential sealing process (2 h for the first sealing and 4 h for the second sealing) using a PDMS stamp rendering 3 μm wide line-shape protruding features. An MCU SAM-covered Pt surface was first disrupted through this process and then immersed in HAuCl_4_ solution for Au nanoparticle generation. For phycocyanin detection, the substrate was immersed in aqueous phycocyanin solutions of various concentrations for 1 h. The obtained image fluorescence intensities were analyzed by the ImageJ software (*N* = 5).

### Real Sample Recovery Tests

For the tap water sample test, the aqueous sample solution was spiked with phycocyanin to a final concentration of 0.2 mg/mL. The AuPt bimetallic substrate was immersed in this spiked solution for 1 h, followed by thorough rinsing with deionized water. This immersion-washing cycle was repeated three times. For the green tea sample test, a commercial green tea (NuLife brand) sample was diluted 100-fold and then spiked with phycocyanin to a final concentration of 0.5 mg/mL. The AuPt bimetallic substrate was immersed in this solution for 1 h and then washed with deionized water. The process was repeated three times as described above.

## Supplementary Material



## Notes

Figure S1: Topographic AFM images and cross-section profiles of patterned features on SAM-functionalized Au; Figure S2: comparison of Au substrate Au 4f XPS spectra before and after siloxane oligomers transportation to Au; Figure S3: optical images and cross-section profiles of etched Au substrates demonstrating the reusability of a PDMS stamp; Figure S4: optical images of Au galvanic replacement on different SAM-disrupted metal substrates; Figure S5: optical images of Pd and Pt substrates after SAM disruption and Au galvanic replacement; Figure S6: SEM images of a MCU (C_11_–OH) SAM-covered Pt substrate after SAM disruption and Au galvanic replacement; Figure S7: SEM, optical, and fluorescence images of AuPt bimetallic substrates prepared using different concentrations of HAuCl_4_; Figure S8: SEM images of AuPt bimetallic substrates prepared using different PDMS sealing times; and Figure S9: scattering cross section of Au nanoparticles on Pt substrates prepared by different PDMS sealing times (PDF)
