# Supplementary material for: Ethanol-Assisted Alkanethiol Self-Assembled Monolayer Disruption by Mobile Siloxane Oligomers for Precise Galvanic Replacement Positioning
Source: ACS Appl Mater Interfaces. 2026 Feb 17;18(8):13184–99. doi: 10.1021/acsami.5c23948 (PMC12964335; doi:10.1021/acsami.5c23948)
Supplement: Supplementary file 1 [file am5c23948_si_001.pdf]

Supporting Information

Ethanol-Assisted Alkanethiol Self-Assembled Monolayer Disruption by Mobile Siloxane Oligomers for Precise Galvanic Replacement Positioning

Yu-Ling Tu,[1] *Chia-Li Liao,*[1]*Elmer Ismael Guerra,*[1] *Heng-Yu Yang,*[1] *Yu-Chieh Wen,*[3] *Lee-Chiang Lo,*[1] *and Wei-Ssu Liao*[1,2,]*

[1]Department of Chemistry, National Taiwan University, Taipei 10617, Taiwan

[2]Center for Emerging Material and Advanced Devices, National Taiwan University, Taipei 10617, Taiwan

[3]Institute of Physics, Academia Sinica, Taipei 11529, Taiwan

*To whom correspondence should be addressed: wsliaochem@ntu.edu.tw (W.S.L.)

Synthesis of d-MCU (4-1)

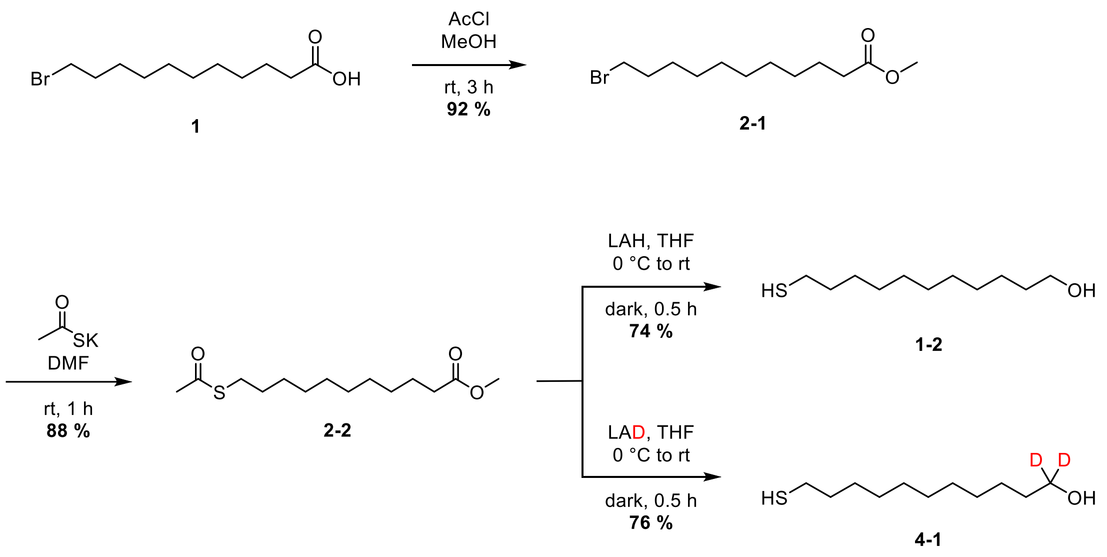

Scheme S1. Synthesis of d-MCU (4-1).

General Considerations for d-MCU Synthesis

All reagents and starting materials were purchased from commercial suppliers (Acros, Fluorochem, Merck, Sigma-Aldrich, Strem, and TCI) and used without further purification. The anhydrous solvents and reagents including acetonitrile (MeCN), dichloromethane (CH_2Cl_2), methanol (MeOH), pyridine (py), and triethylamine (Et_3N) were distilled with calcium hydride powder. Anhydrous tetrahydrofuran (THF) was distilled with metallic sodium and benzophenone. *N*,*N*-Dimethylformamide (DMF) was treated with 4 Å molecular sieves overnight before use.

Molecular sieves were activated by heating at 250 °C for 8 h under vacuum. Reactions were monitored with analytical thin-layer chromatography (silica gel, 60 F_{254}, Merck) and visualized under UV light or stained with cerium molybdate (Hanessian's stain), phosphomolybdic

acid (PMA) in ethanol, 5% potassium permanganate ($KMnO_4$) with 5% potassium carbonate in 1 M NaOH $_{(aq)}$, or 5% sulfuric acid in ethanol.

Column chromatography was performed with 230-400 mesh Kiesegel 60 silica gel (Merck), while reverse phase chromatography was performed with C_{18} silica gel (LC-Sorb, SP-S-ODS, manufactured by Chemco corporation). Mobile phases are reported in the ratio of solvents for binary systems (e.g. Hexane/EtOAc = 9/1).

Characterizations in d-MCU Synthesis

Nuclear magnetic resonance (NMR) spectra were recorded on a Bruker AVIII (^{1}H: 400 MHz; ^{13}C: 100 MHz) spectrometer at room temperature. Chemical shifts (*d*) are given in parts per million (ppm) relative to d_H 7.26 / d_C 77.2 for $CDCl_3$, d_H 3.31 / d_C 49.0 for MeOD-d_4, d_H 2.50 / d_C 39.5 for DMSO-d_6, and d_H 4.79 for D_2O. Coupling constant (*J*) are given in hertz (Hz) and the splitting patterns are designated by the following abbreviations: s, singlet; d, doublet; t, triplet; q, quartet; quin, quintet; m, multiplet; and br, broad. Raw fid files were processed by Bruker TopSpin 4.3.0 software.

Infrared spectroscopy (IR) spectra were recorded on a Varian 640 FT-IR instrument by applying the sample onto a KBr plate (neat) to prepare pellets and the absorption data are reported in cm^{-1}.

Electrospray ionization high-resolution mass spectrometry (ESI-HRMS) data were obtained using a Bruker micrOTOF-QII or JEOL JMS-700 instrument.

Melting points were recorded using a Fischer Scientific melting point apparatus and all samples were recorded without correction.

NMR Spectra of Synthesized Compounds

^{1}H-NMR spectrum of compound **2-1** (400 MHz, $CDCl_3$)

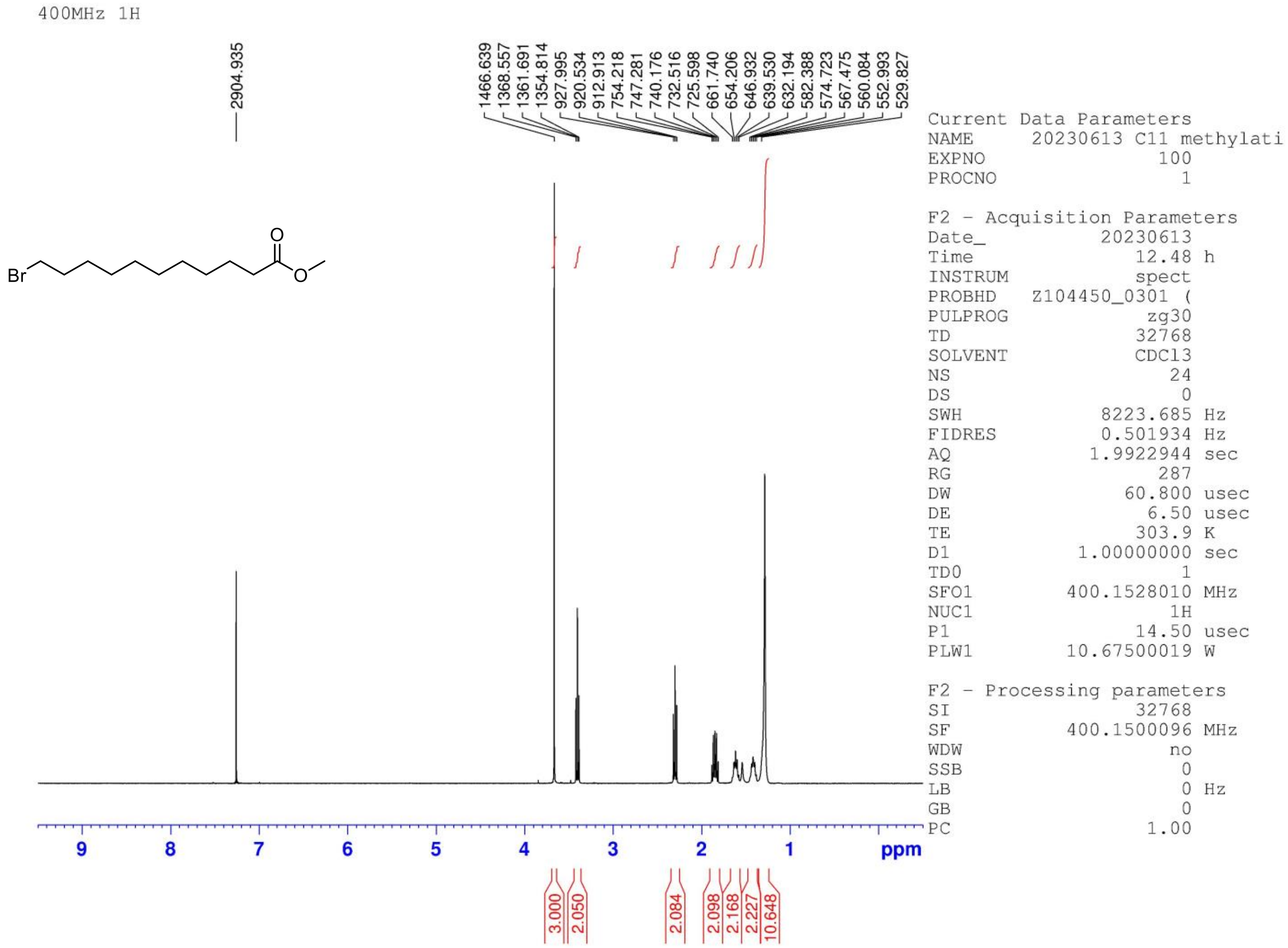

^{1}H-NMR spectrum of compound **2-2** (400 MHz, $CDCl_3$)

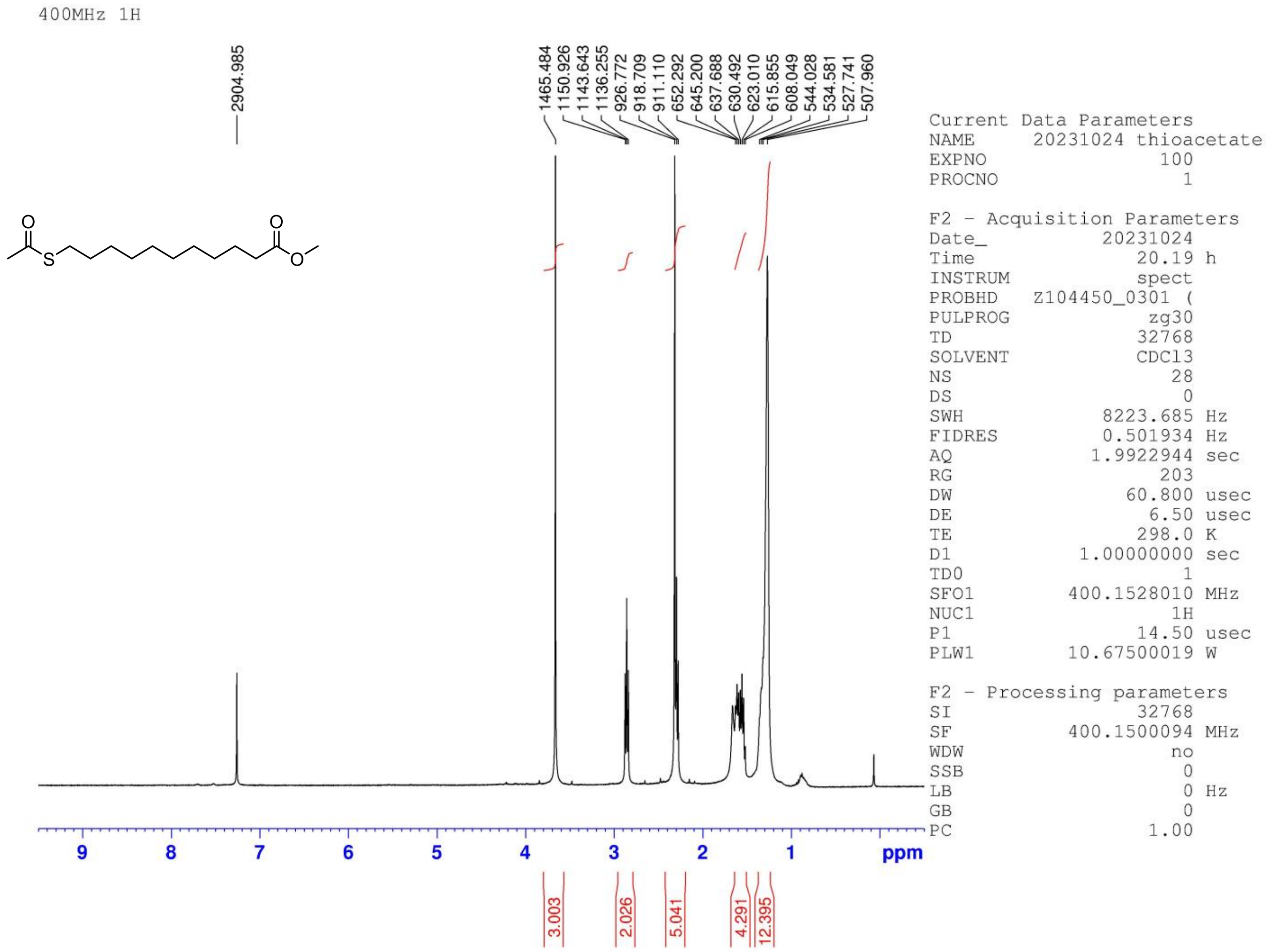

^{1}H-NMR spectrum of compound **1-2** (400 MHz, $CDCl_3$)

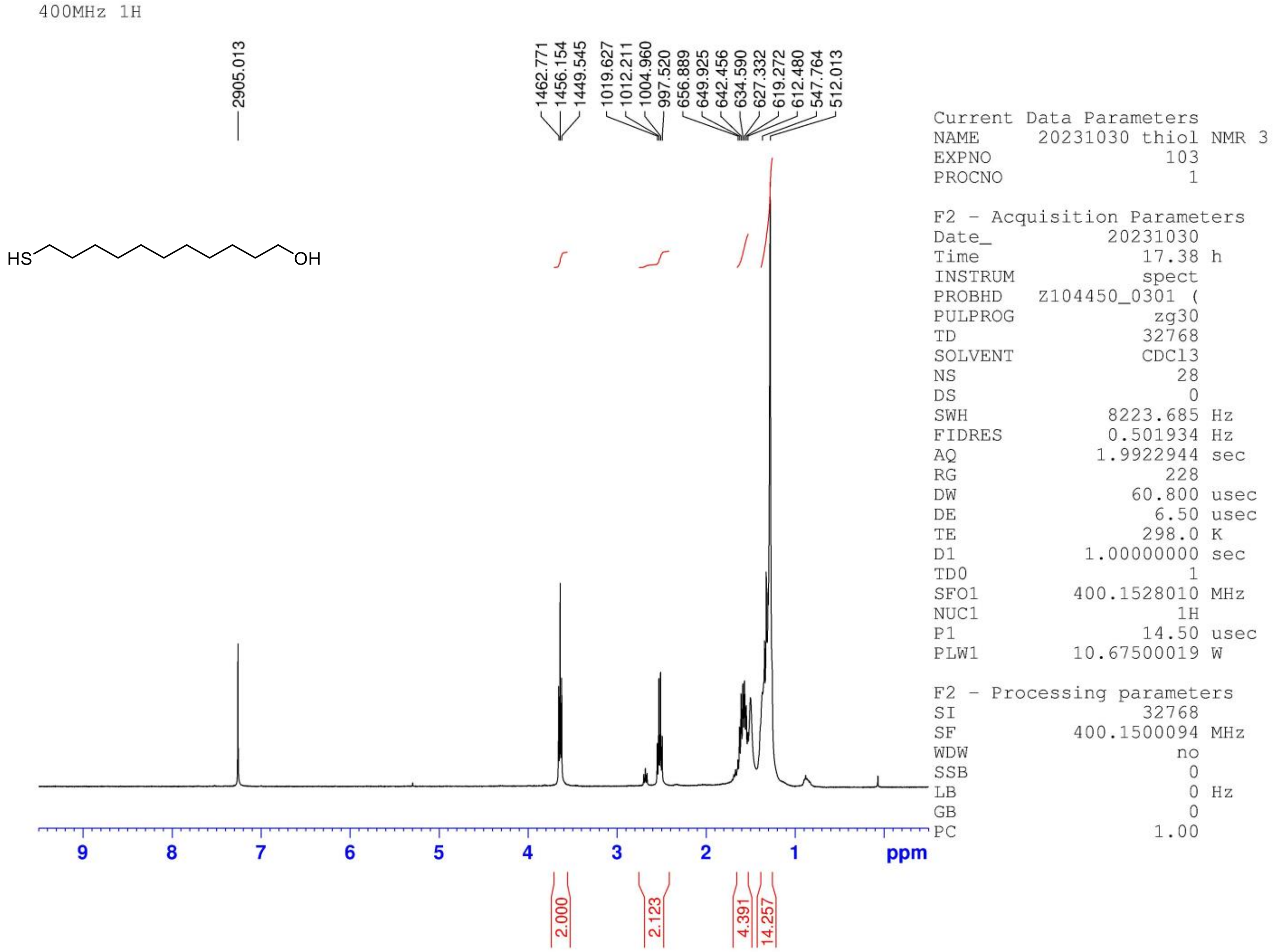

^{13}C-NMR spectrum of compound **1-2** (100 MHz, $CDCl_3$)

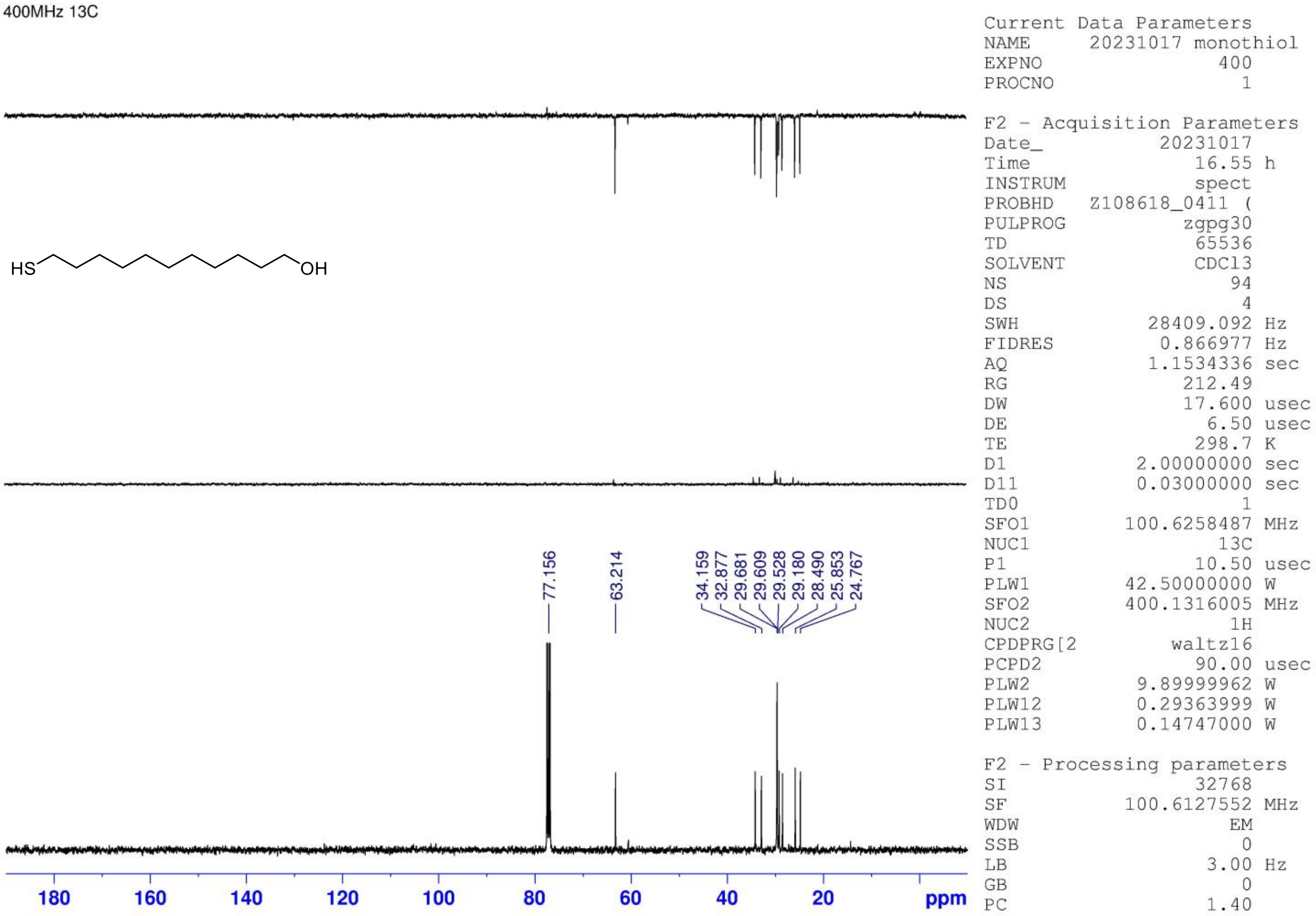

^{1}H-NMR spectrum of compound **4-1** (400 MHz, $CDCl_3$)

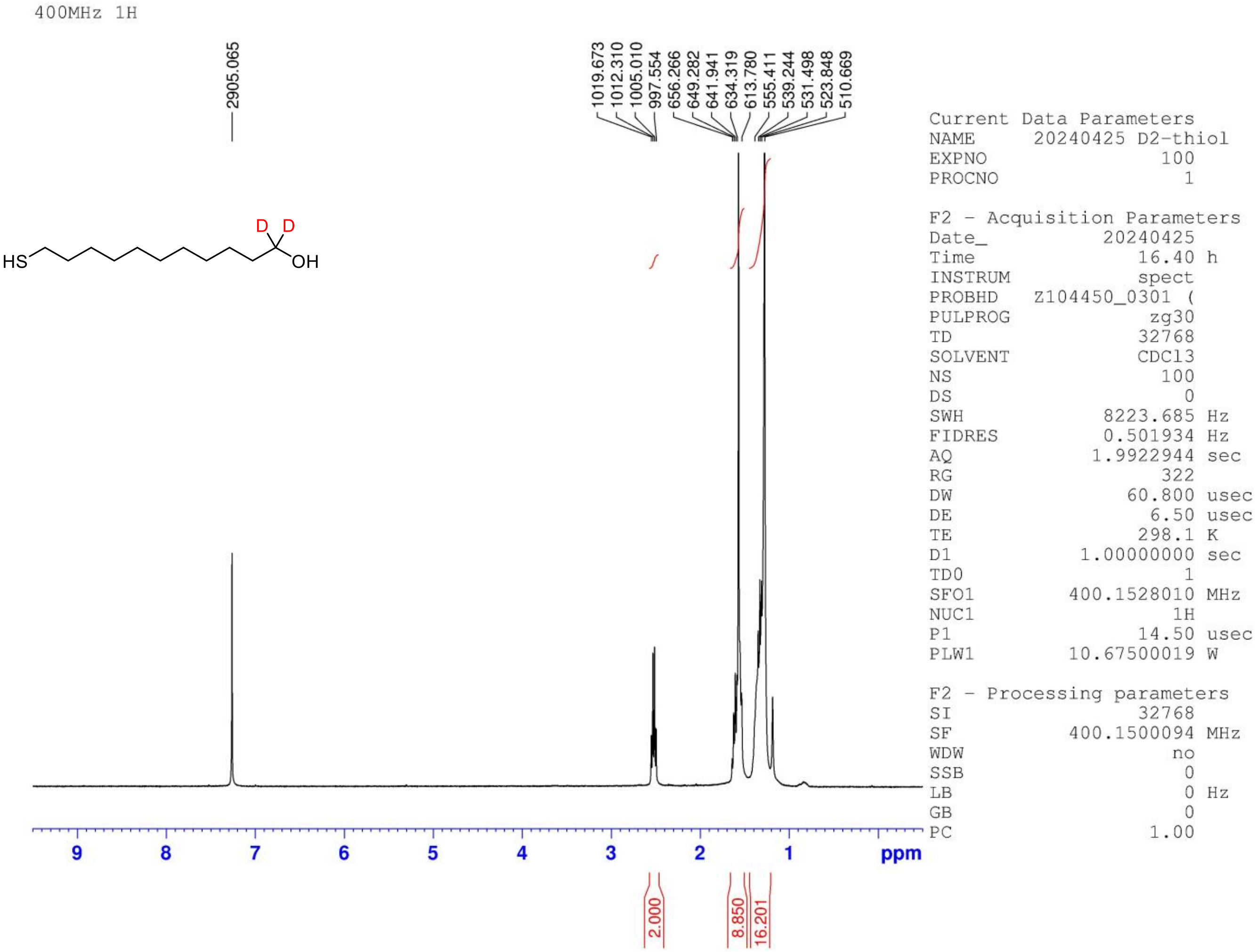

^{13}C-NMR spectrum of compound **4-1** (100 MHz, $CDCl_3$)

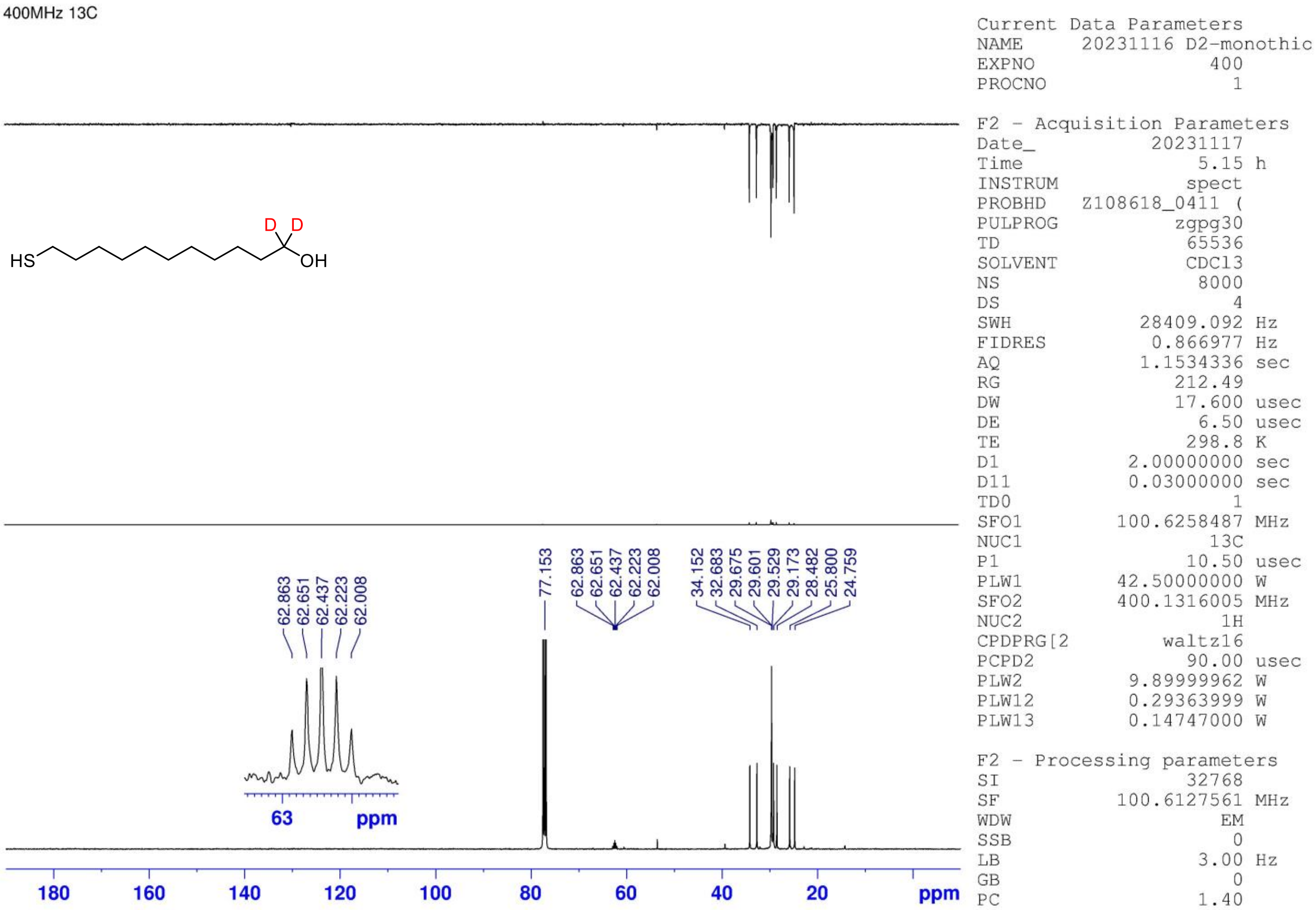

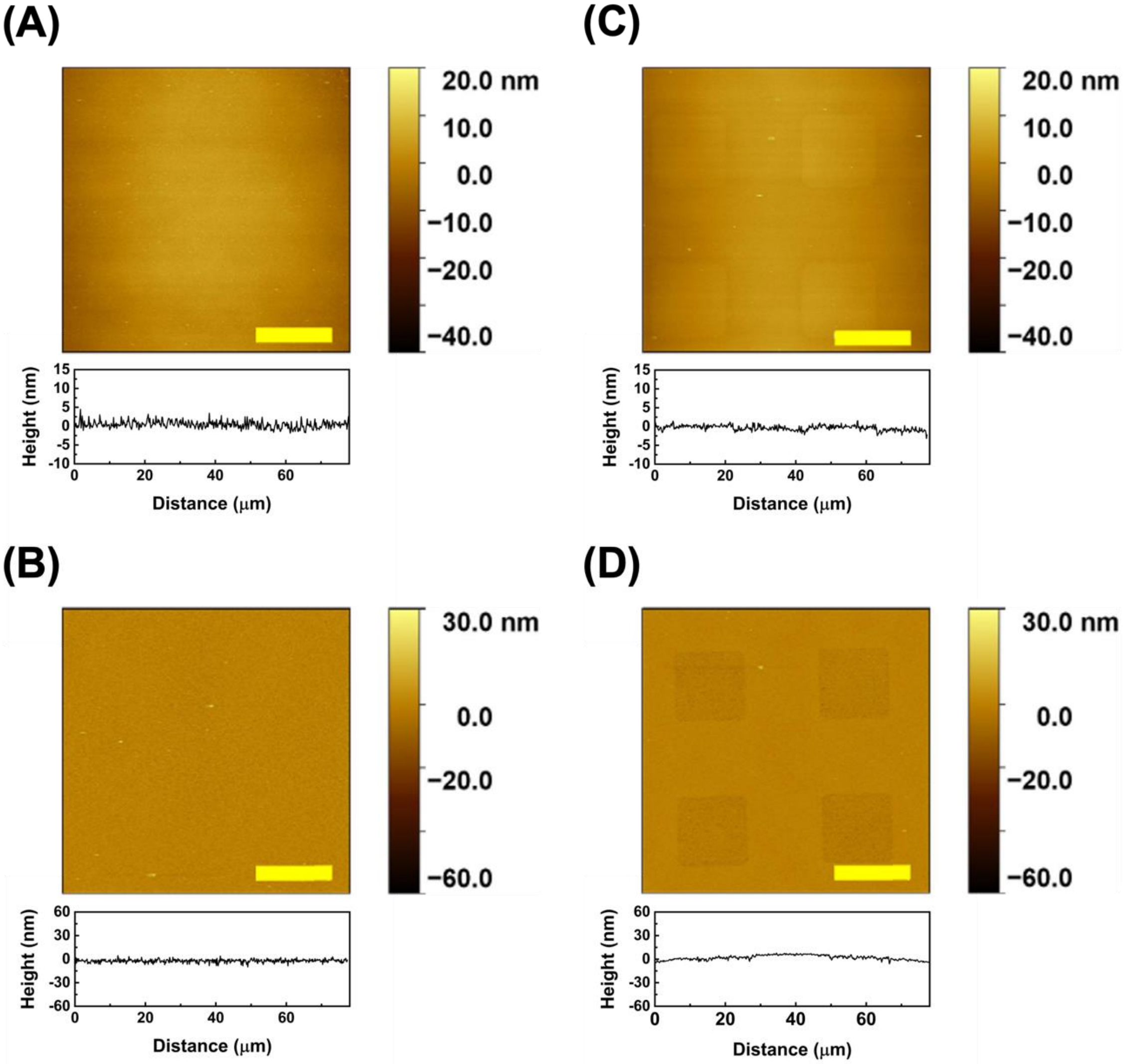

Figure S1. Topographic AFM images and cross-section profiles detailing the extent of SAM disruption on SAM-functionalized Au substrates fabricated by ethanol-assisted siloxane oligomer insertion using PDMS stamps with 20 μm protruding square features. The PDMS stamps were pre-soaked in ethanol for 30 min. Upper experimental conditions: PDMS conformal sealing for (A) 1 h and (B) 3 h before Au etching. Lower experimental conditions: PDMS conformal sealing for (C) 1 h and (D) 3 h after Au etching. Scale bars are 20 μm.

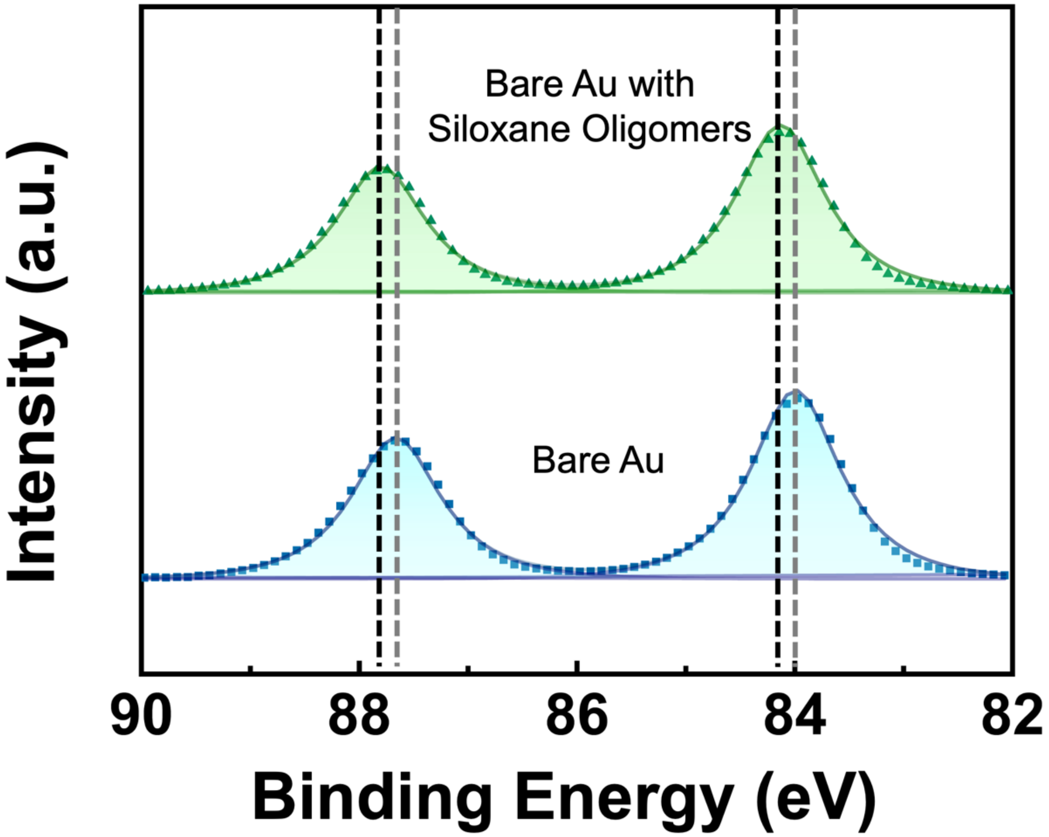

Figure S2. Comparison of Au substrate Au 4f XPS spectra before and after siloxane oligomers transportation to Au. The black dashed line indicates the binding energy of bare Au with siloxane oligomers, while the gray dashed line indicates the original binding energy of bare Au.

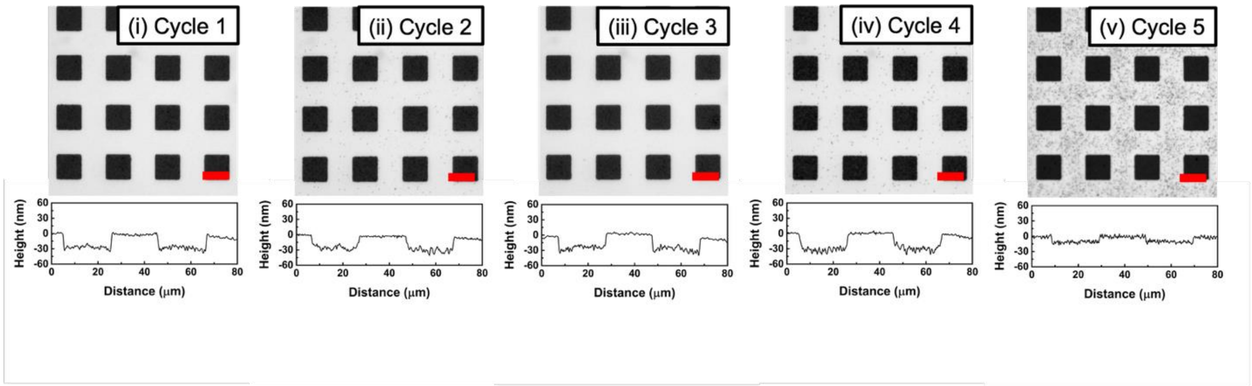

Figure S3. Optical images and cross-section profiles of etched Au substrates demonstrating the reusability of a PDMS stamp over multiple uses, from (i) the first time to (v) the fifth application, employing the ethanol-assisted siloxane oligomer insertion-induced SAM disruption. Scale bars are 20 μm.

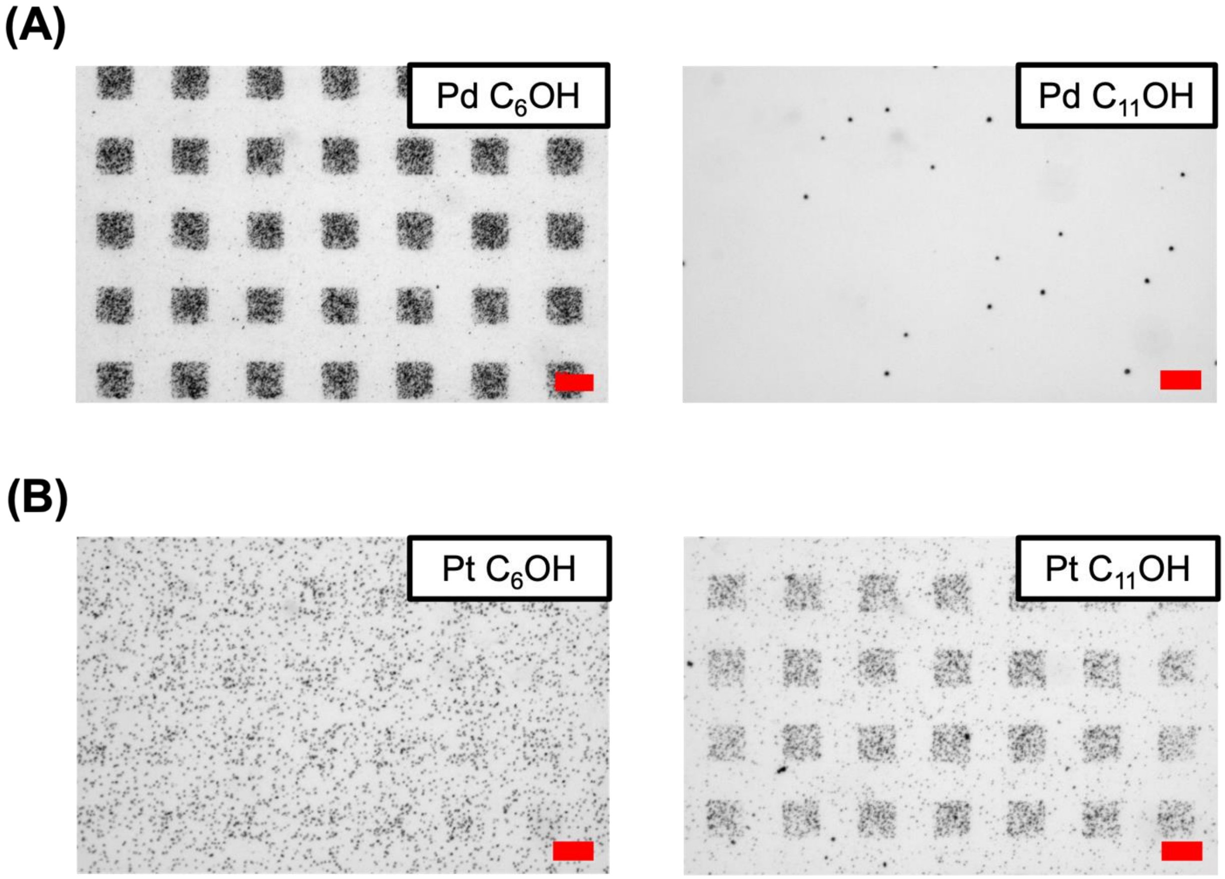

Figure S4. Optical images of Au galvanic replacement on different SAM-disrupted metal substrates demonstrating the effect of alkanethiol backbone length. (A) Pd substrates modified with MCH (C_6OH) (left) and MCU ($C_{11}OH$) (right), respectively. (B) Pt substrates modified with MCH (C_6OH) (left) and MCU ($C_{11}OH$) (right), respectively. Scale bars are 20 μm.

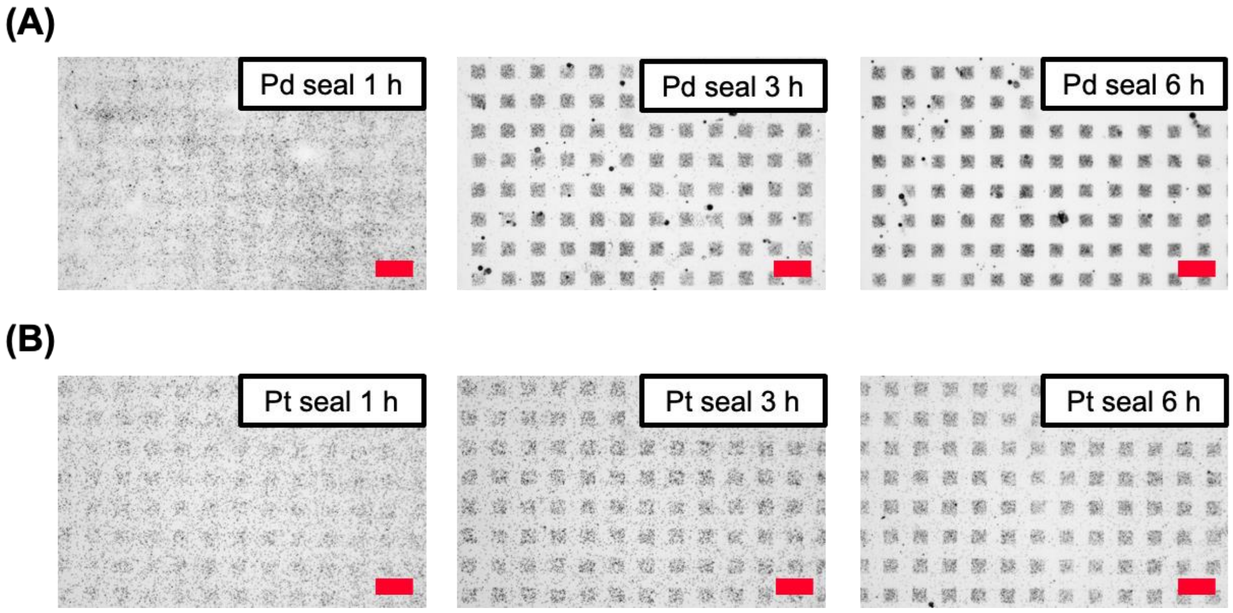

Figure S5. Optical images of (A) Pd substrates functionalized with MCH (C_6OH) and (B) Pt substrates modified with MCU ($C_{11}OH$) following SAM disruption and Au galvanic replacement. From left to right: 1 h, 3 h, and 6 h of PDMS conformal sealing in the SAM disruption process. Scale bars are 50 μm.

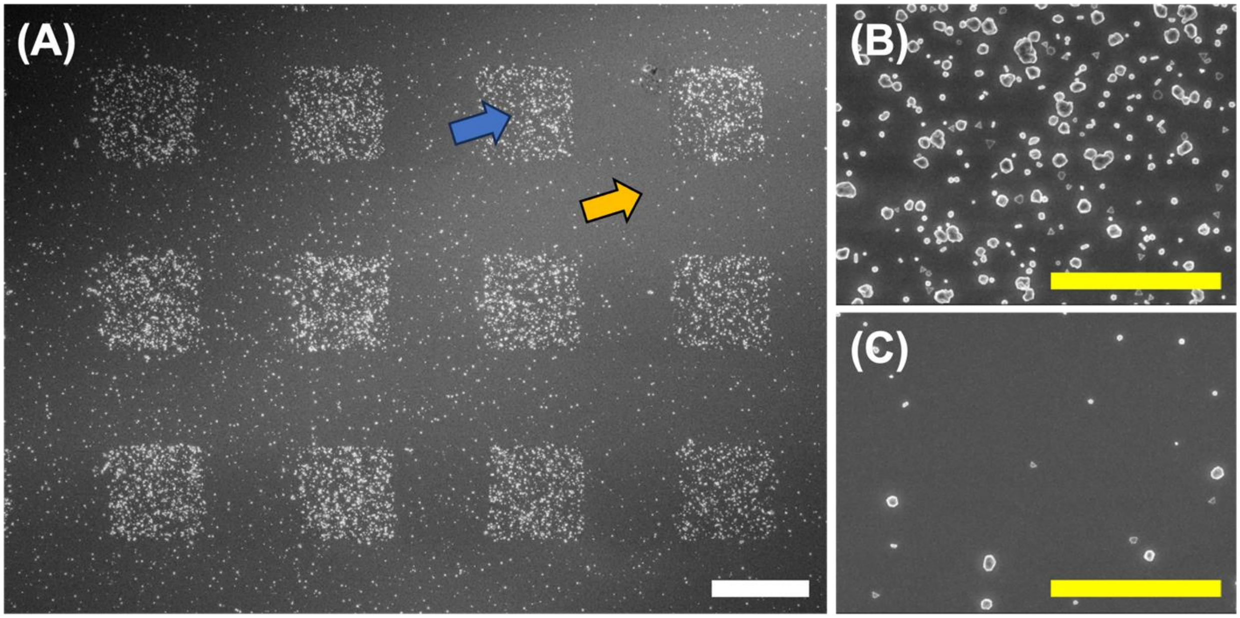

Figure S6. (A) SEM images of a MCU ($C_{11}OH$) SAM-covered Pt substrate after ethanol-assisted SAM disruption and Au galvanic replacement. A PDMS stamp with 20 µm protruding square features is used for the conformal sealing process. Blue and orange arrows indicate the inside (B) and outside (C) of the PDMS contact region. White scale bar is 20 µm and yellow scale bars are 5 µm.

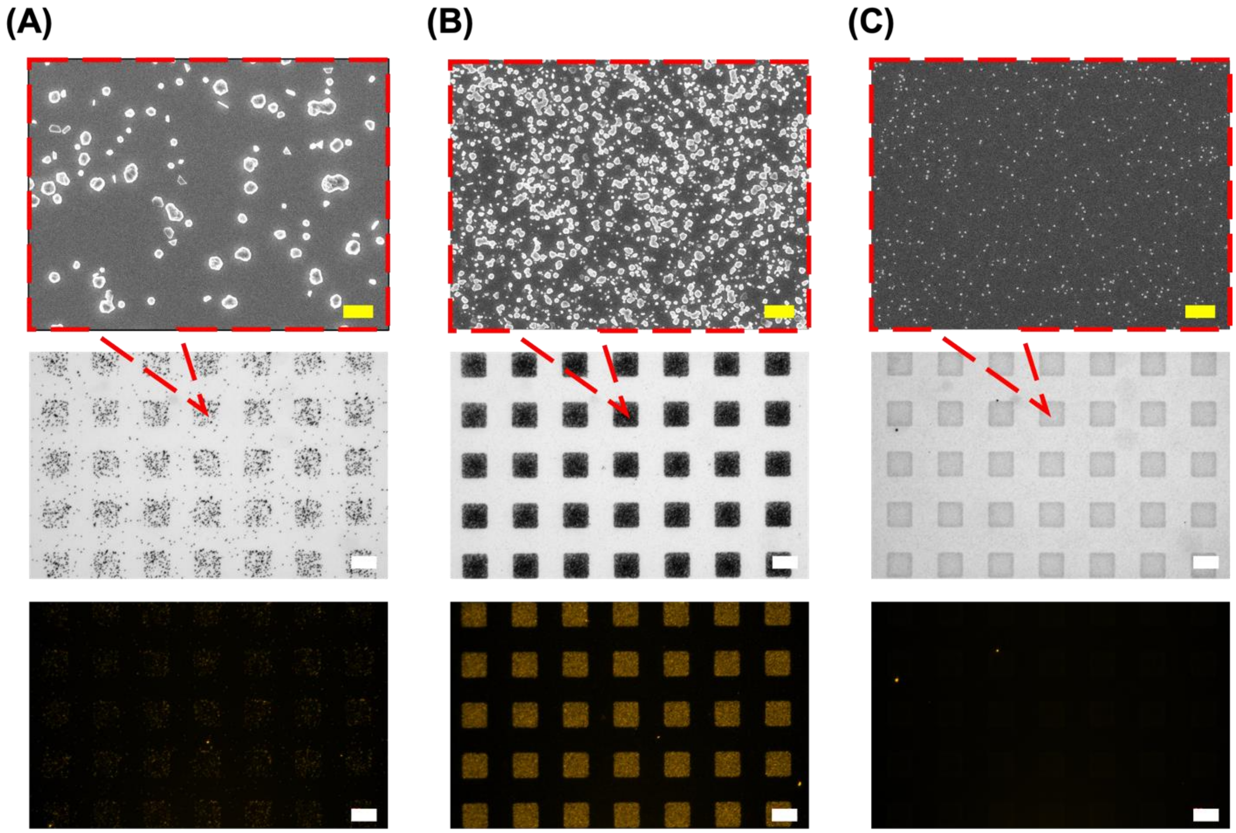

Figure S7. (Top) SEM images, (middle) optical images, and (bottom) fluorescence images of AuPt bimetallic substrates prepared using different concentrations of $HAuCl_4$: (A) 1 mM, (B) 0.1 mM, and (C) 0.01 mM. For the fluorescence images, R6G dye was used to monitor the MEF effect. The yellow scale bars are 1 μm and the white scale bars are 20 μm.

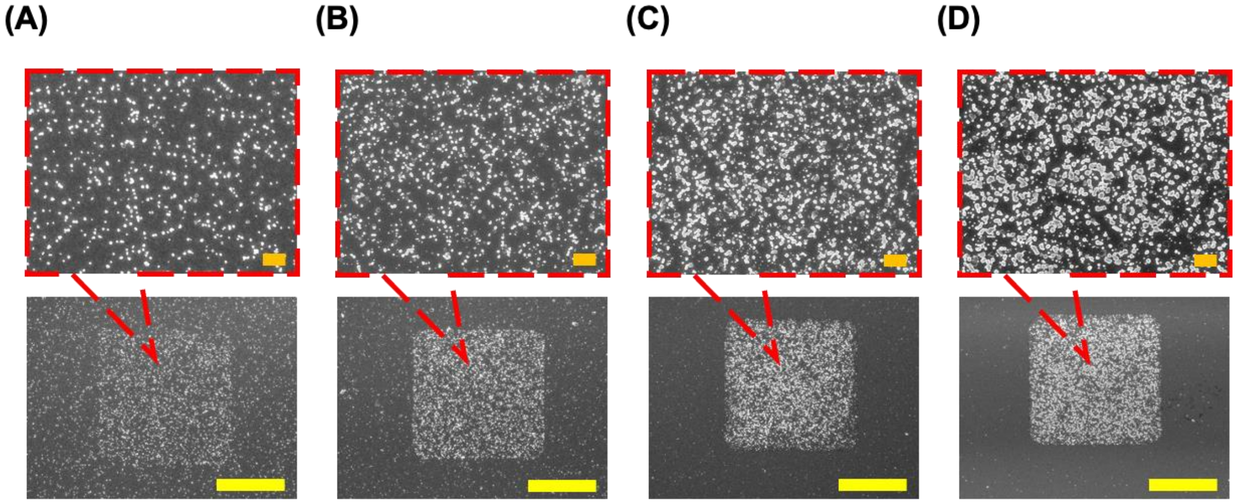

Figure S8. SEM images of AuPt bimetallic substrates prepared using different PDMS sealing times: (A) 0.5 h, (B) 1 h, (C) 3 h, and (D) 6 h. The orange scale bars are 1 μm and the yellow scale bars are 10 μm.

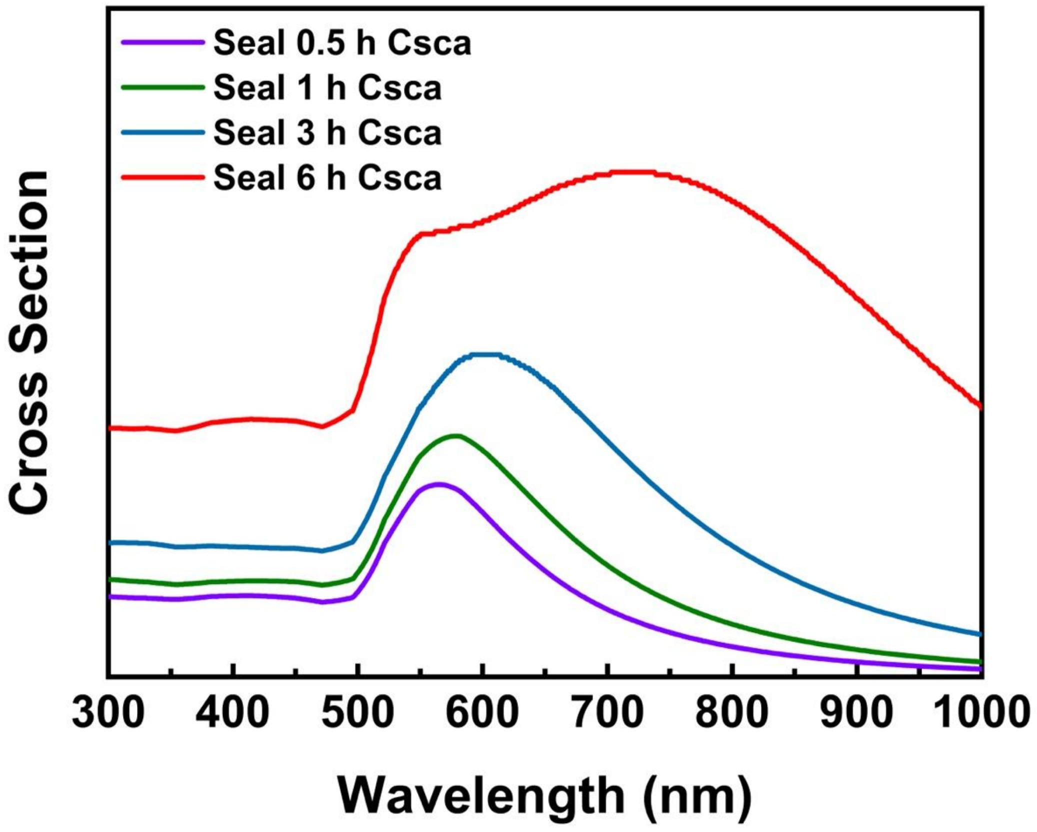

Figure S9. Scattering cross-section of Au nanoparticles on Pt substrates prepared by different PDMS sealing times.
